# Discovery of New Pyrazolopyridine, Furopyridine, and Pyridine Derivatives as CDK2 Inhibitors: Design, Synthesis, Docking Studies, and Anti-Proliferative Activity

**DOI:** 10.3390/molecules26133923

**Published:** 2021-06-26

**Authors:** Adel A.-H. Abdel-Rahman, Amira K. F. Shaban, Ibrahim F. Nassar, Dina S. EL-Kady, Nasser S. M. Ismail, Samy F. Mahmoud, Hanem M. Awad, Wael A. El-Sayed

**Affiliations:** 1 Chemistry Department, Faculty of Science, Menofia University, Shbien El-Kom 32511, Egypt; Adel.nassar@science.menofia.edu.eg; 2 Faculty of Specific Education, Ain Shams University, Abassia, Cairo 11772, Egypt; Dr.ibrahim.nassar@sedu.asu.edu.eg; 3 Hormone Department, National Research Centre, Dokki, Cairo 12622, Egypt; dn_science@yahoo.com; 4 Pharmaceutical Chemistry Department, Faculty of Pharmacy, Future University in Egypt, Cairo 12311, Egypt; Nasser.saad@fue.edu.eg; 5 Department of Biotechnology, College of Science, Taif University, P.O. Box 11099, Taif 21944, Saudi Arabia; S.Farouk@tu.edu.sa; 6 Tanning Materials and Leather Technology Department, National Research Centre, Dokki, Cairo 12622, Egypt; Hanem_awad@yahoo.com; 7 Department of Chemistry, College of Science, Qassim University, Buraidah 51452, Saudi Arabia; 8 Photochemistry Department, National Research Centre, Dokki, Cairo 12622, Egypt

**Keywords:** pyridine, imidazole, pyrazolo[3,4-b]pyridine, anticancer, HepG2, docking, CDK2

## Abstract

New pyridine, pyrazoloyridine, and furopyridine derivatives substituted with naphthyl and thienyl moieties were designed and synthesized starting from 6-(naphthalen-2-yl)-2-oxo-4-(thiophen-2-yl)-1,2-dihydropyridine-3-carbonitrile (**1**). The chloro, methoxy, cholroacetoxy, imidazolyl, azide, and arylamino derivatives were prepared to obtain the pyridine-*^−^C*^2^ functionalized derivatives. The derived pyrazolpyridine-*N*-glycosides were synthesized via heterocyclization of the *C*^2^-thioxopyridine derivative followed by glycosylation using glucose and galactose. The furopyridine derivative **14** and the tricyclic pyrido[3′,2′:4,5]furo[3,2-d]pyrimidine **15** were prepared via heterocyclization of the ester derivative followed by a reaction with formamide. The newly synthesized compounds were evaluated for their ability to in vitro inhibit the CDK2 enzyme. In addition, the cytotoxicity of the compounds was tested against four different human cancer cell lines (HCT-116, MCF-7, HepG2, and A549). The CDK2/cyclin A2 enzyme inhibitory results revealed that pyridone **1**, 2-chloro-6-(naphthalen-2-yl)-4-(thiophen-2-yl)nicotinonitrile (**4**), 6-(naphthalen-2-yl)-4-(thiophen-2-yl)-1*H*-pyrazolo[3,4-b]pyridin-3-amine (**8**), S-(3-cyano-6-(naphthaen-2-yl)-4-(thiophen-2-yl)pyridin-2-yl) 2-chloroethanethioate (**11**), and ethyl 3-amino-6-(naphthalen-2-yl)-4-(thiophen-2-yl)furo[2,3-b]pyridine-2-carboxylate (**14**) are among the most active inhibitors with IC_50_ values of 0.57, 0.24, 0.65, 0.50, and 0.93 µM, respectively, compared to roscovitine (IC_50_ 0.394 μM). Most compounds showed significant inhibition on different human cancer cell lines (HCT-116, MCF-7, HepG2, and A549) with IC_50_ ranges of 31.3–49.0, 19.3–55.5, 22.7–44.8, and 36.8–70.7 μM, respectively compared to doxorubicin (IC_50_ 40.0, 64.8, 24.7 and 58.1 µM, respectively). Furthermore, a molecular docking study suggests that most of the target compounds have a similar binding mode as a reference compound in the active site of the CDK2 enzyme. The structural requirements controlling the CDK2 inhibitory activity were determined through the generation of a statistically significant 2D-QSAR model.

## 1. Introduction

The cyclin-dependent kinases (CDK) (a serine/threonine kinase family) [1] are responsible for the initiation and the succession of each cell cycle phase; CDK2 activity is required for progression through G1 to the S phase [2]. The activity of members of the CDK family is corrupted in many tumor cells where they are essential for the phosphorylation of key components for cell proliferation [3,4]. CDK2 has an important catalytic role in the complex of cyclin-dependent protein kinase; its activity is essential for cell cycle progress [5]. The cyclin protein family modulates CDK activities throughout the cell cycle where specific activating phosphorylation for CDK apoenzymes is required through CDK-activating kinase, which is a CDK complex, and also, complex formation with cyclins is needed for optimum kinase activity [5,6]. CDK2 is associated mainly with the regulatory subunits including either cyclin A or E with overexpression in human cancer as in ovarian, breast, endometrial, lung and thyroid carcinomas, osteosarcoma, and melanoma [7]. The activation of the CDK2 through cyclin A is claimed to be an essential step through the progression from the S phase, and this makes CDK2 the principal target for most inhibitory drugs [3,4,5,6].

**Figure 1:**
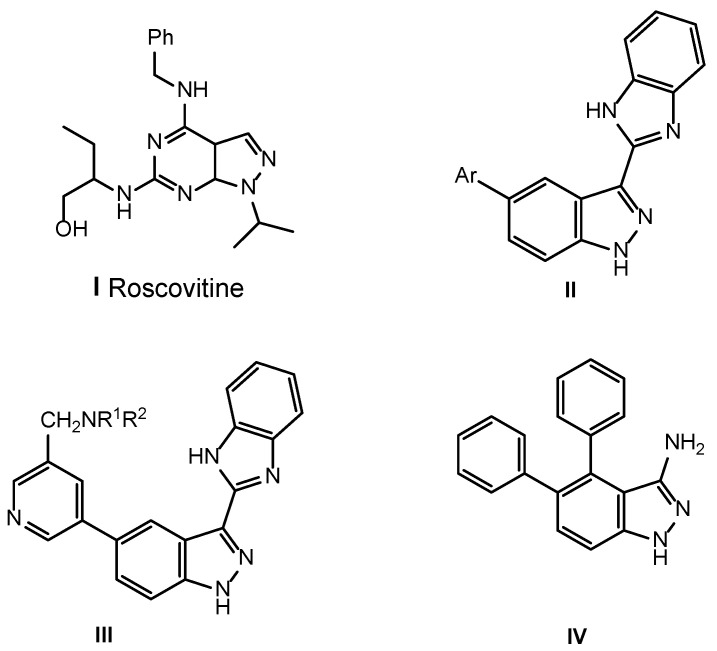
Roscovitine, (pyridin-3-yl)-1H-indazole and imidazolyl-1*H*-indazole derivatives with potent inhibition activity for CDK2.

Heterocyclic ring systems have emerged as powerful scaffolds for many biological evaluations [8] and play an important role in the design and discovery of new physiological/pharmacologically active molecules [9]. The incorporation of more than one heterocyclic core in one hybrid structure, known as molecular hybridization, gained considerable interest in the design and synthesis of bioactive compounds [10,11,12]. The pyridine core is found in a variety of biologically active compounds possessing a broad spectrum of bioactivities [13,14]. The pyrazolo[3,4-b]pyridine framework is a promising heterocyclic scaffold that has gained renewed interest displaying a broad spectrum of biological activities such as antitumor inhibiting CDK2 [15,16,17,18,19] and glycogen synthase kinase-3 (GSK-3) inhibitors [20]. In addition, pyrazolo [3,4-b]pyridine derivatives represent important building blocks in both natural and synthetic bioactive compounds [21]. Moreover, fused heterocyclic-containing pyrazolopyridine systems have been described as associated with several biological and medicinal activities [22,23,24,25]. Oxygen heterocycles exhibit diverse biological and pharmacological activities due to the similarities with many natural and synthetic molecules [26]. Figure 1 illustrates some important pyrazolopyridine-based compounds with potent activities for CDK2 enzyme [27,28] and its bicyclic congener *Roscovitine*, which is a CDK2 inhibitor undergoing clinical trials to treat cancers [29].

Furo[2,3-b]pyridine/pyrido[30,20:4,5]furo[3,2-d]pyrimidine with different substitutions has gained renewed interest as a template for drug discovery. Furo[2,3-b]pyridine derivatives also demonstrated in vitro activity for tubulin polymerization [30] as well as Lck [31] and Akt [32] kinase inhibitors. Imidazole and thiophene-containing compounds showed various bioactivities, the most important of which is anticancer [33,34,35]. On the other hand, glycoside and their analogs have been revealed as an important bioactive group of compounds with anticancer, antiviral, and antimicrobial activity. The therapeutic importance of this scaffold motivated us to develop selective procedures for the synthesis of new derivatives of pyridine incorporating pyrazolyl, imidazolyl, thienyl, and glycosyl moieties in which substituents could be arranged in a pharmacophoric pattern to display high order of anticancer activity [36,37,38,39,40,41,42,43].

## 2. Results and Discussion

### 2.1. Chemistry

The ease synthesis and high biological activity of the starting 6-(naphthalen-2-yl)-2-oxo-4-(thiophen-2-yl)-1,2-dihydropyridine-3-carbonitrile (**1**) prompted us to prepare some of its derivatives starting from available and inexpensive compounds. The reaction of compound **1** with chloroacetyl choloride yielded 3-cyano-6-(naphthalen-2-yl)-4-(thiophen-2-yl)pyridin-2-yl-2-chloroacetate (**2**) via substitution reaction in 84% yield. The structure of compound **2** was elucidated based on elemental and spectral analyses. The IR spectrum of 2 revealed strong absorption bands at ν 2216 and 1697 cm^−1^ for CN and CO groups, respectively. The ^1^H-NMR of the same compound showed signals at *δ* 4.40 ppm referring to CH_2_Cl in addition to the signals of the aromatic protons. The formation of imidazolthione **3** was obtained through the heterocyclization of compound **2** by reaction with thiourea in DMF. Its IR spectrum showed the disappearance of the absorption band at ν 1697 cm^−1^ of the carbonyl group and the appearance of the characteristic bands at ν 3424 and 1260 cm^−1^ due to NH and C=S groups, respectively. The ^1^H-NMR spectrum of compound **3** exhibited very distinct singlet signals resonating at δ 4.13 (imidazole-H) and δ 12.98 (exchangeable NH) confirmed the successful formation of 3. ^13^C-NMR spectrum of compound **3** showed signals at δ 49.6, 168.1, and 188.4 ppm for imidazoles-C and C=S groups, respectively beside the aromatic carbons at their specific regions (see supplementary materials).

**Scheme 1:**
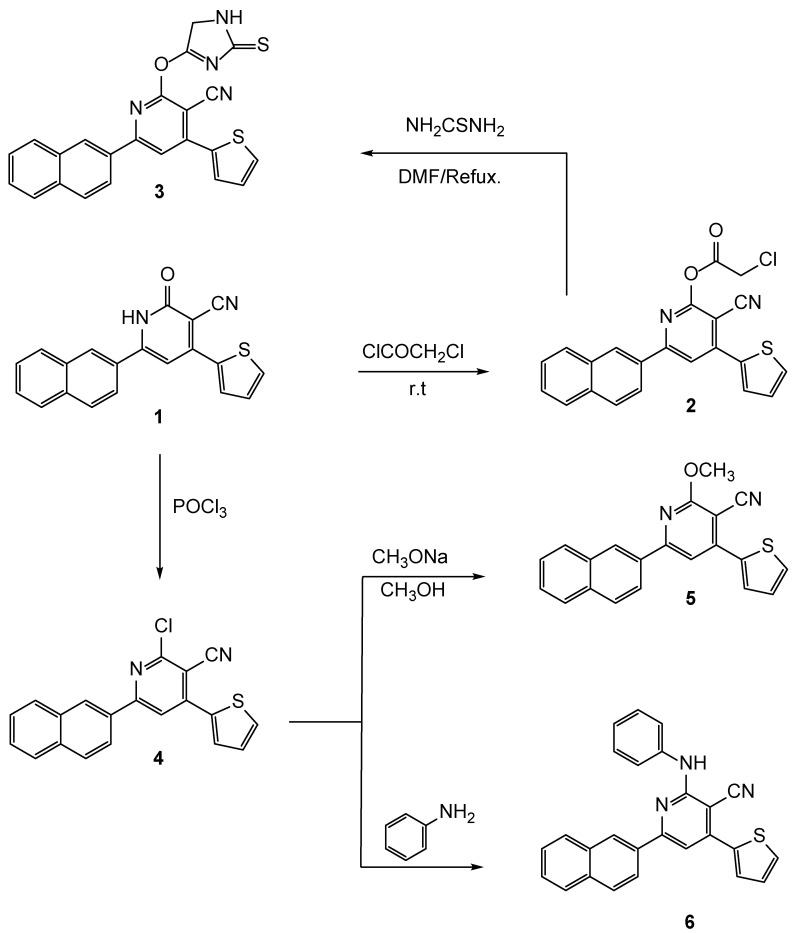
Synthesis of substituted pyridine derivatives (2, 4-6) and (imidazolyl)oxynicotinonitrile derivatives 3.

Compound **4** [44] underwent different substitution reactions. First, compound **4** was allowed to react with sodium methoxide in methanol to give 2-methoxy-6-(naphthalen-2-yl)-4-(thiophen-2-yl)nicotinonitrile (**5**) in 75% yield. Its ^1^H-NMR revealed a signal at δ 4.16 ppm due to methoxy protons. In addition, compound **4** was reacted with aniline to give 6-(naphthalen-2-yl)-2-(phenylamino)-4-(thiophen-2-yl)nicotinonitrile (**6**). IR spectrum of 6 revealed the presence of a strong absorption band at *ν* 3428 cm^−1^ due to the NH group, and also, it appeared as a strong signal at δ 9.80 ppm in the ^1^H-NMR spectrum (Scheme 1).

On the other hand, pyridine-2(1H) thione derivative 7 [45] was reacted with hydrazine hydrate in ethanol under reflux conditions to afford 3-amino-1H-pyrazolo[3,4-b]pyridine derivatives (**8**) in a good yield. The IR spectrum of 8 revealed bands at ν 3375–3228 cm^−1^ referred to NH_2_ and NH groups. The ^1^H-NMR exhibited a singlet signal at δ 6.45 ppm referring to the exchangeable NH_2_ protons and also a singlet signal at δ 11.65 ppm corresponding to the exchangeable NH proton. ^13^C-NMR spectra agreed with such investigation and confirmed their structures, which showed that C-pyrazoles have been revealed at δ 90.2, 151.3, and 152.2 ppm.

Compound **8** was further reacted with the monosaccharides such as *d*-glucose or *d*-galactose as six-carbon sugars in ethanol, and a few drops of glacial acetic acid afforded compounds **9** and **10**, respectively, which have been shown to incorporate sugar moieties in the cyclic form. Their IR spectra showed strong and broad absorption bands characterizing the polyhydroxy chain and NH groups in the range ν 3520–3390 cm^−1^. The ^1^H-NMR spectra of the same compounds showed signals for NH (D_2_O exchangeable) and OH groups at their specific regions (see supplementary materials).

The reaction proceeds according to the proposed mechanism [43].

**Scheme 2:**
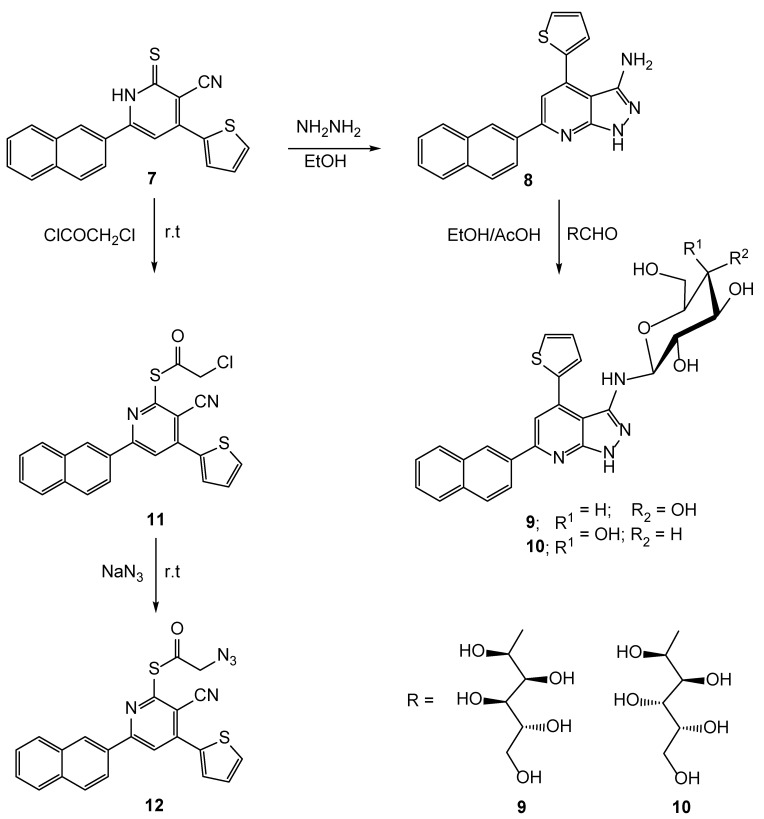
Synthesis of pyrazolopyridine-N-glycosides (9 and 10) and pyridinyl-2- azidoethanethioate derivative 12.

In addition, compound **11** was synthesized by stirring compound **7** with chloroacetyl chloride in acetone and anhydrous potassium carbonate at room temperature. The IR spectrum of **11** showed the appearance of CH aliphatic, C=O and CN, bands at ν 2921, 1698, and 1590 cm^−1^, respectively. The ^1^H-NMR spectrum of **11** showed a sharp singlet signal at *δ* 4.30 ppm belonging to the CH_2_Cl protons beside the singlet signal at *δ* 8.19 ppm for pyridine proton, thus confirming the formation of compound **11**. The azido compound **12** was produced when compound **11** was stirred at room temperature with sodium azide in *N*, *N*-dimethyl formamide. The IR spectrum of compound **12** revealed strong absorption bands assigning the N_3_ at ν 2366 cm^−1^. The ^1^H-NMR spectra of that compound showed a characteristic singlet signal for CH_2_N_3_ at *δ* 2.42 ppm in addition to aromatic protons. ^13^C-NMR spectra of compound **12** revealed signals at *δ* 65.1 and 191.0 ppm attributed to CN_3_ and CO groups respectively beside the aromatic carbons at their specific region (Scheme 2).

**Scheme 3:**
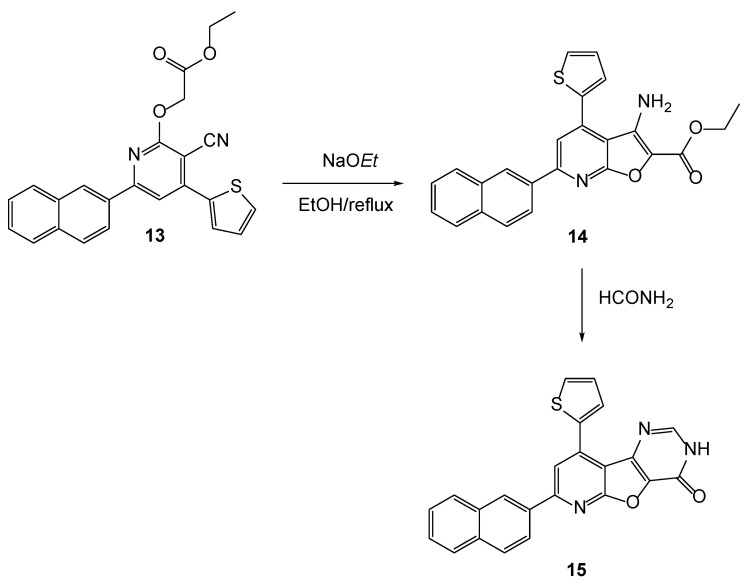
Synthesis of substituted pyrido[3′,2′:4,5]furo[3,2-d]pyrimidin-4(3H)-one derivative 15.

The ester **13** was refluxed with sodium ethoxide to give ethyl 3-amino-6-(naphthalen-2-yl)-4-(thiophen-2-yl)furo[2,3-b]pyridine-2-carboxylate (**14**). This compound might be subjected to further structural modification. The formation of the furan ring with two functionalities (the amino and the carboethoxy groups) resulted, whose further modifications could provide the opportunity for new cyclization processes. The IR spectrum of **14** showed absorptions bands at *ν* 3437 cm^−1^ referring to the NH_2_ group and a strong absorption band at *ν* 1739 cm^−1^ referring to the ester carbonyl group. The ^1^H-NMR spectrum exhibited a singlet signal of the amino group at *δ* 5.61 ppm, in addition to triplet and quartet signals at *δ* 1.2 and 4.3 ppm for CH_3_ and CH_2_ groups. Compound **14** was condensed with electrophiles namely, formamide, to afford the pyridofuropyrimidine **15**. The chemical structure of **15** was elucidated by its correct elemental analysis and spectral data. Its IR spectrum showed absorption bands at *ν* 3419 and 1662 cm^−1^ for the imino (NH) and the amidic carbonyl group (C=O). The ^1^H-NMR spectrum revealed singlet signals at *δ* 8.26 and 8.34 ppm for pyridine and pyrimidine protons, multiplet signals in the range of δ 7.24–8.11 ppm for aryl protons, and a single signal at *δ* 11.18 corresponding to the imine proton NH (D_2_O exchangeable) (Scheme 3).

### 2.2. Biological Evaluation

#### 2.2.1. CDK2/Cyclin A2 Activity

The in vitro CDK2/cyclin A2 assays of the newly synthesized compounds **1**–**5**, **7**, **8**, **11**, **13**, and **14** were carried out using Promega Kinase-Glo Plus luminescence kinase kit. It measures kinase activity by quantitating the amount of ATP remaining in solution following a kinase reaction. The luminescent signal from the assay is correlated with the amount of ATP present and is inversely correlated with the amount of kinase activity. The percent inhibition of the tested compounds against CDK2/cyclin A2 protein kinase was compared to reference kinase inhibitor roscovitine at a single concentration of 10 μM. The computer software Graphpad Prism was used for analyzing the luminescence data. The difference between luminescence intensities in the absence of CDK2/cyclin A2 protein kinase (Lut) and in the presence of kinase (Luc) was defined as 100% activity (Lut − Luc). Using luminescence signal (Lu) in the presence of the compound, the percentage of activity was calculated as: % activity = {(Lut − Lu)/(Lut − Luc)} × 100%, where Lu = the luminescence intensity in the presence of the compound. The percentage of inhibition was calculated as: % inhibition = 100 (%) − % activities [46].

The IC_50_ results of the target compounds are presented in Table 1. All the new compounds revealed a potent inhibitory effect against CDK2/cyclin A2 protein kinase with an IC_50_ values ranging from 3.52 to 0.24 μM compared to roscovitine reference compound of IC_50_ 0.39μM. Amongst them, compound **4** revealed the highest IC_50_ of 0.24 μM more potent than roscovitine, while compounds **1**, **8**, **11**, and **14** showed good inhibitory activities comparable to the reference compound with IC_50_ 0.57, 0.65, 0.50, and 0.93 μM, respectively. Moreover compounds **2**, **3**, **5**, **7**, and **13** revealed promising activity with IC_50_ values ranging 1.01–4.45 μM.

#### 2.2.2. In Vitro Cytotoxicity Activity

**Figure 2:**
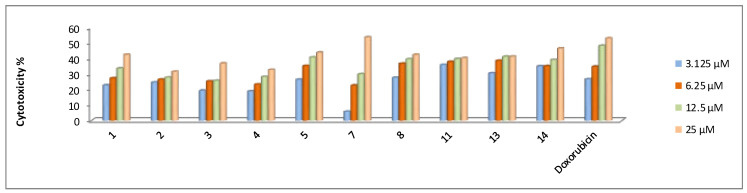
Dose-dependent cytotoxic activities of the compounds against HCT-116 cancer cells according to the MTT assay.

**Figure 3:**
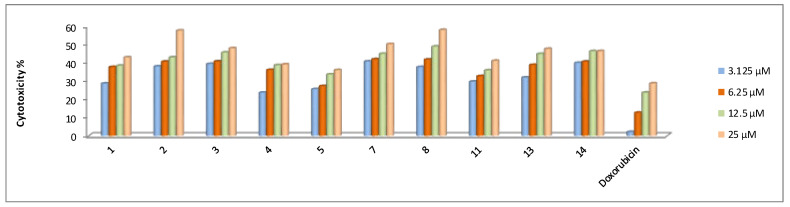
Dose-dependent cytotoxic activities of the compounds against MCF-7 cancer cells according to the MTT assay.

**Figure 4:**
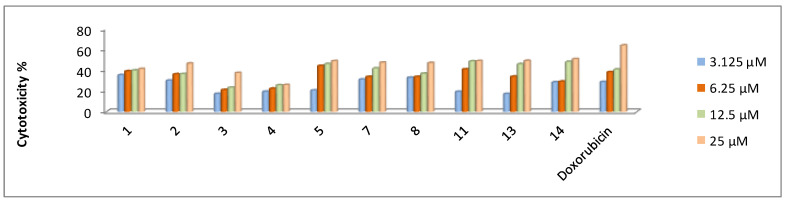
Dose-dependent cytotoxic activities of the compounds against HepG2 cancer cells according to the MTT assay.

**Figure 5:**
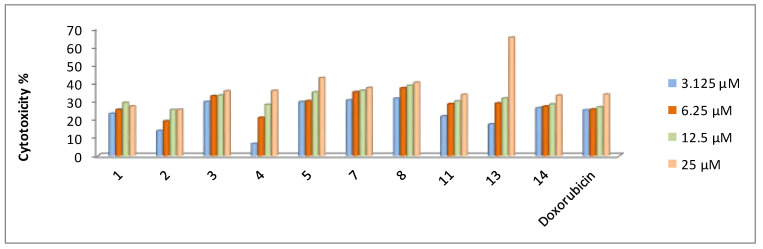
Dose-dependent cytotoxic activities of the compounds against A549 cancer cells according to the MTT assay.

In this study, ten compounds were examined in vitro for their activities against HCT-116, MCF-7, HepG2, and A549 human cancer cells using the MTT assay. The percentages of intact cells were calculated and compared to those of the control. The activities of these compounds against the four cell lines were compared to the activity of doxorubicin as well. All compounds suppressed the four human cells in a dose-dependent manner (Figure 2, Figure 3, Figure 4 and Figure 5). In order to study the efficacy of the synthesized compounds, a comparison of the cytotoxic effect of each compound has been related to the cytotoxicity of the reference drug as follows.

In case of HCT-116 human colorectal carcinoma cells, both Figure 2 and Table 2 show that six compounds (**14**, **11**, **13**, **8**, **5**, and **7** respectively) have insignificantly more potent anticancer activities; compound **1** has equipotent anticancer effect to doxorubicin; three compounds (**2**, **4** and **3**, respectively) exert a smaller anticancer effect compared to that of doxorubicin.

However, all the screened compounds (**1**–**5**, **7**, **8**, **11**, **13**, and **14**) showed higher inhibitory activity compared to doxorubicin (IC_50_ = 64.8 µM) against MCF-7 (Figure 3 and Table 2).

Meanwhile, only compound **7** has more potent anticancer activity against HepG2 with IC_50_ 22.7 µM, compared to that of the reference compound (Figure 4 and Table 2).

For A549 human lung cancer cells, seven compounds (**7**, **8**, **13**, **5**, **14**, **11**, and **3**, respectively) have better anticancer activities; the rest of the compounds have insignificantly less anticancer activity compared to that of doxorubicin (Figure 5 and Table 2).

#### 2.2.3. Molecular Docking Study

**Figure 6:**
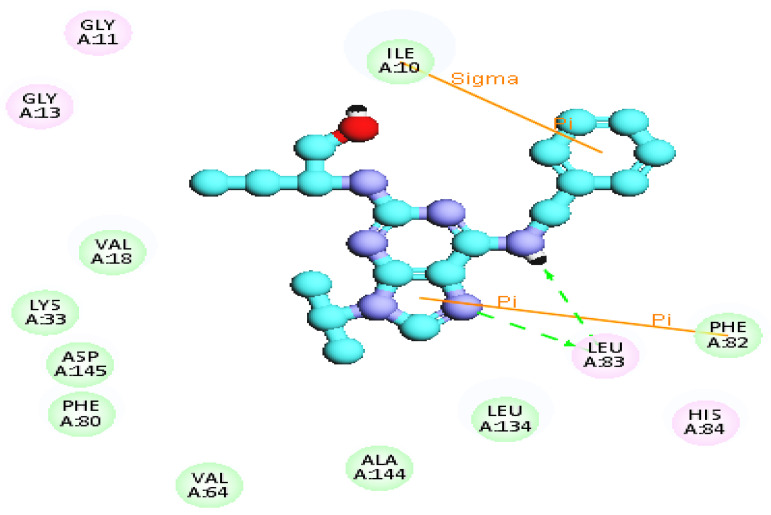
Two-dimensional (2D) diagram of roscovitine in the active site of CDK2. Dot green line represents the hydrogen bond donor and acceptor. Orange color line represents the hydrophobic interaction.Amino acids with green color represent the Van der Waals interaction, while the purple amino acids represent the electrostatic interaction.

**Figure 7:**
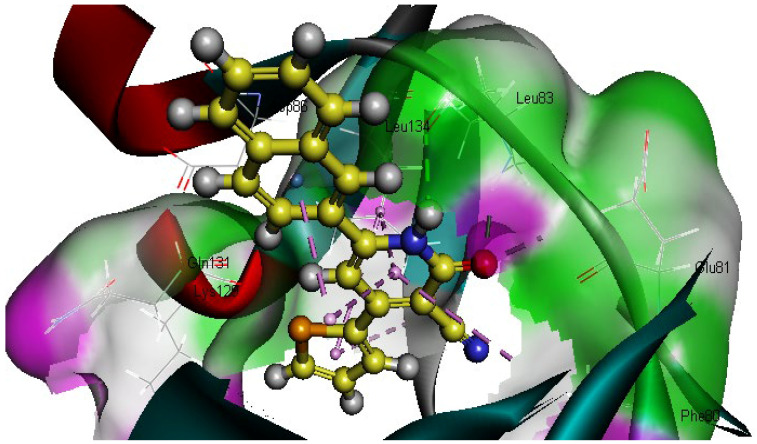

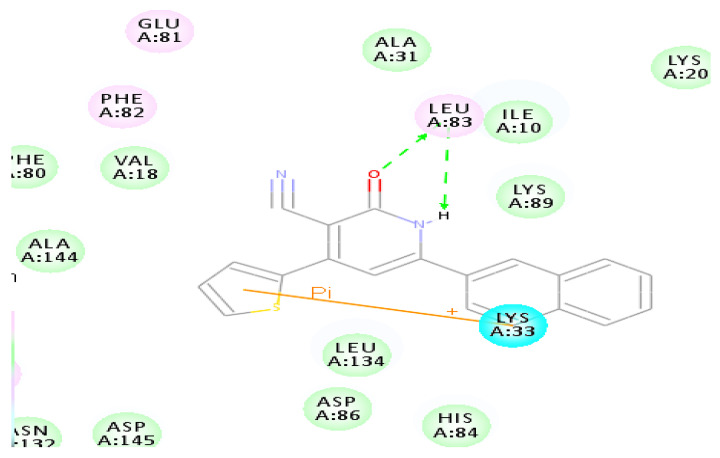
Two-dimensional (2D) and 3D docking of compound **1** into the CDK2 active site. Amino acids with green color represent the Van der Waals interaction; the purple amino acids represent the electrostatic interaction, while the blue amino acid represents the hydrophobic interaction.

**Figure 8:**
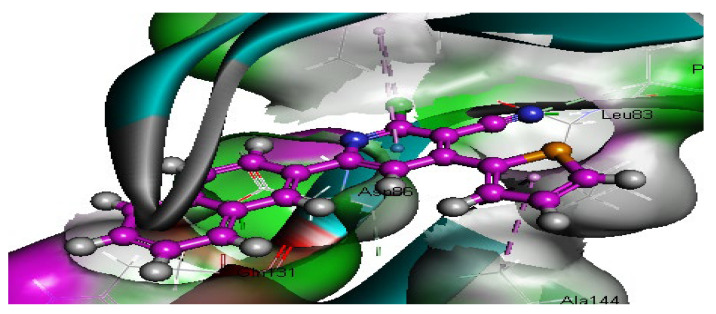
Docking of compound **4** into the CDK2 active site. Purple color represent carbon atoms, gray color represent hydrogen atom, blue color represent nitrogen atom, orange color represent sulfur atom and green color represent chlorine atom.

**Figure 9:**
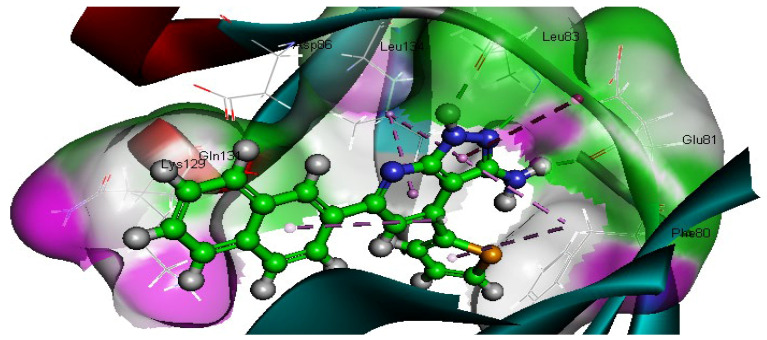

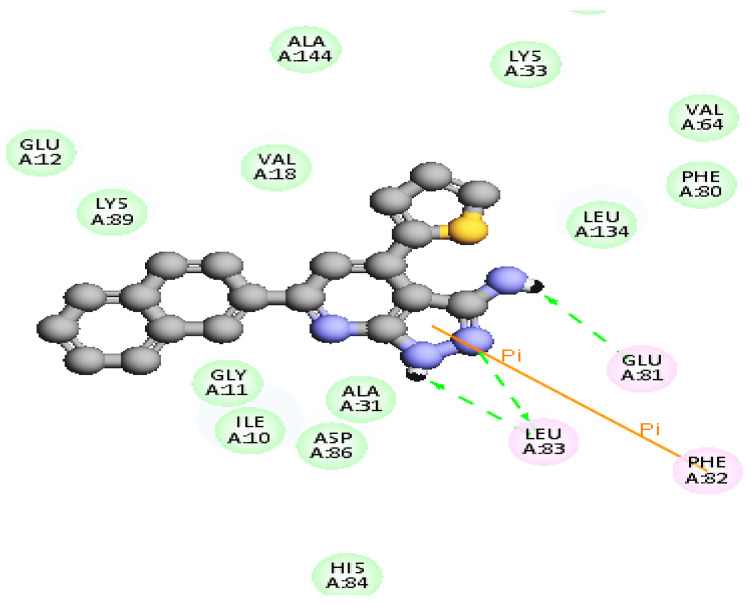
Docking of compound **8** into the CDK2 active site. Gray color represent carbon atoms, blue color represent nitrogen atoms, while the orange color represent sulfur atoms.

**Figure 10:**
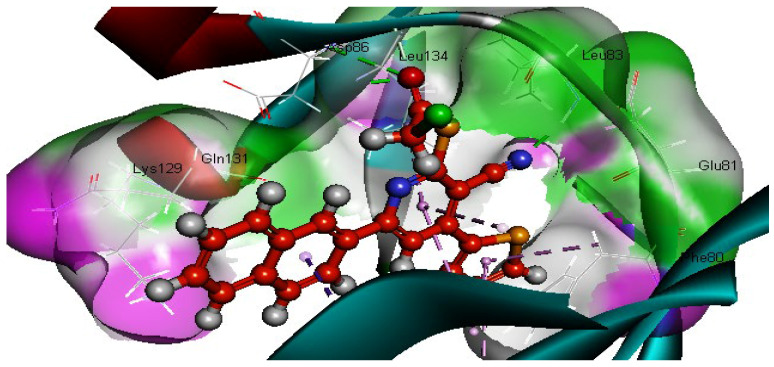
Docking of compound **11** into the CDK2 active site. Red color represent the carbon atoms of molecules, gray color represent hydrogen atoms, the blue color represent the nitrogen atoms, the orange color represent sulfur atom and The green color represent chlorine atom.

**Figure 11:**
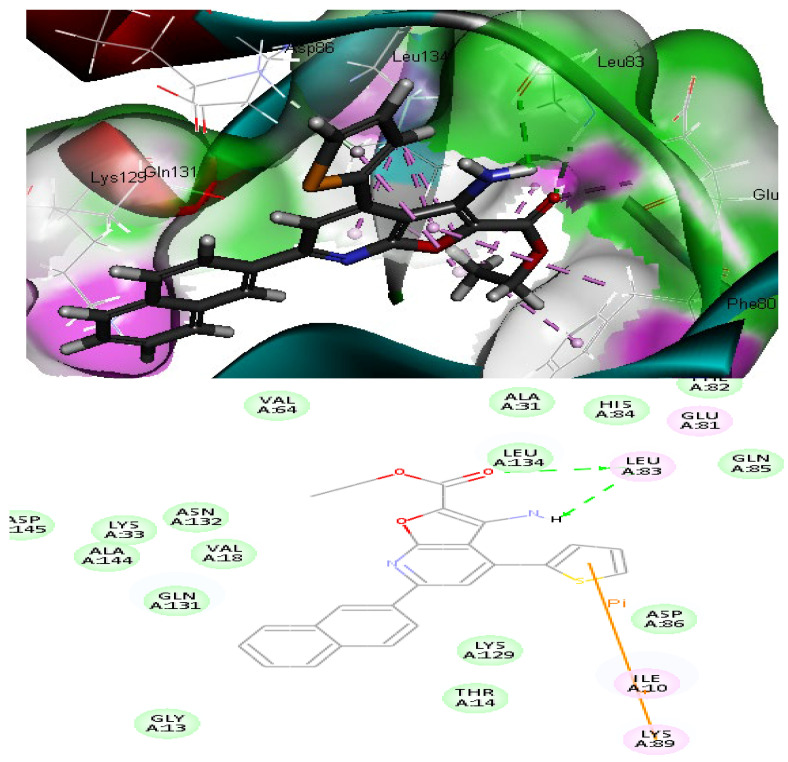
Docking of compound **14** into the CDK2 active site.

The molecular simulation study was carried out through docking of the target compounds in the binding pocket of the CDK2 enzyme using C-Docker protocol in Discovery Studio 4.0 Software. In this work, molecular docking study and analysis of the binding modes for the designed compounds were achieved to interpret the biological results and to gain further insight into orientations and interactions with the key amino acids. It has been found that both the X-ray crystallographic enzyme (CDK2) substrate complex with roscovitine (PDB code 2A4L) [47] revealed the formation of the essential two H-bonds with Leu83. Validation of the C-Docker protocol in this investigation was performed by re-docking of the co-crystallized lead compound in the CDK2 kinase active site. The calculated RMSD value (RMSD = 0.46 A°) indicated the ability of the used docking protocol to retrieve valid docking poses. The docking of the target compounds under study, into the active site of the CDK2 enzyme, revealed comparable binding modes to the lead compound. The binding interactions of the docked compounds together with their binding energies and the key amino acids involved are presented in Table 3 and Figure 6, Figure 7, Figure 8, Figure 9, Figure 10 and Figure 11.

**Figure 12:**
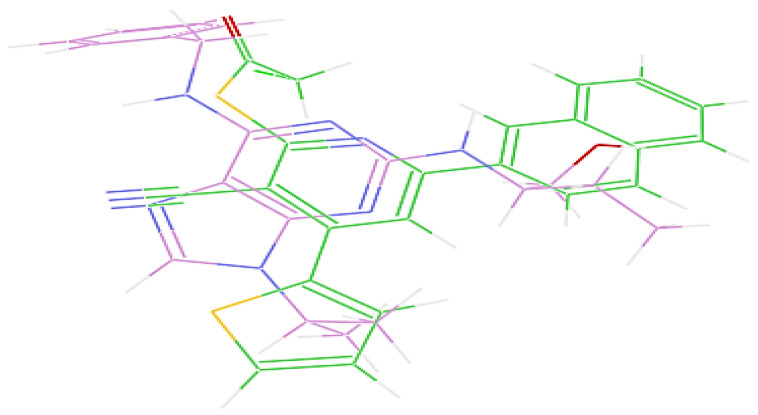
Alignment of compound **11** (green) with roscovitine (purple).

By exploring the C-Docker interaction energy and the binding mode of the highly biologically active compounds **1**, **4**, **8**, **11**, and **14**, it was found that compounds **1**, **4**, **11**, and **14** conserved the formation of the two H bonds with Leu83 as the reference compound (roscovitine), while compound **8** showed one H bond with Leu83 and another additional two H bonds with Asp86 (Figure 7, Figure 8, Figure 9, Figure 10 and Figure 11). These compounds (**1**, **4**, **8**, **11**, and **14**) exhibited high antitumor activity against cancer cell lines (HCT-116, MCF-7, HepG2, A549) with high inhibitory activities against the CDK2 enzyme. Whereas compounds **2**, **3**, **5**, **7**, and **13** showed low binding energy compared to that of the lead compound with the formation of only one H bond with Leu83 and compounds **2**, **5**, and **7** formed an additional H bond with Lys89. Additional interaction was found between most of the docked compounds and Lys89, Lys33, Phe82, and Ile10. In addition, an alignment study was performed for compound **11**, which showed good alignment with approximately the same pharmacophoric elements of the reference compound (roscovitine) (Figure 12). The alignment study revealed that (i) the purine ring system of the compound roscovitine was perfectly aligned with the purine 3-cyano pyridine moiety of compound **11**, (ii) a thiocarboxamide side chain superimposed with N-benzyl substituent of roscovitine, and (iii) additionally, thiophene moiety and naphtyl group were aligned with N-isoprpyl and N(1-hydroxy-but-2-yl) of roscovitine, respectively.

**Figure 13:**
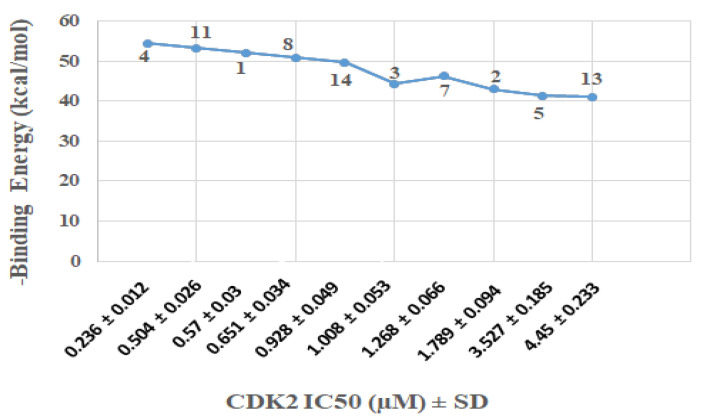
Correlation between the binding energy and the inhibitory profile of the investigated molecules.

Correlation between the binding energy and the inhibitory activity of the investigated molecules showed that the most potent pyridine derivatives **4**, **11**, and **1** (IC_50_ values of 0.24 ± 0.01, 0.50 ± 0.03, and 0.57 ± 0.03 μM, respectively) showed the highest binding energy (Figure 13). Whereas compounds with low IC_50_ values (high potent) revealed high binding energy with –ve (high docking score, more stable in binding site), while compounds with a high IC_50_ (less potent) revealed low binding energy (less docking score). This consistent relation resulted in the successful designing of promising analogs with potent CDK2 inhibitory activity.

##### D-QSAR Studies

**Figure 14:**
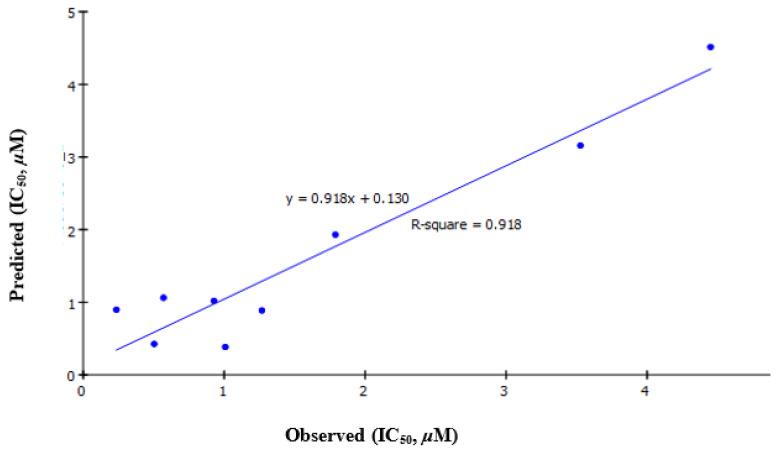
MLR-QSAR model plot of correlations representing the observed versus predicted IC50 μM values for the compounds **1**–**5**, **7**, **11**, **13**, and **14** against the CDK2 enzyme.

Two-dimensional (2D)-QSAR studies were undertaken utilizing Discovery Studio 4.0 Software employing nine bio-active compounds (**1**–**5**, **7**, **11**, **13**, and **14**) as a training set, which exhibit variable inhibitory properties against the CDK2 enzyme. The “Create Multiple Linear Regression Model” protocol was used to generate the 2D-QSAR model predicting the controlled descriptors as statistically significant. The two descriptors controlling the QSAR model are presented in the following equation. Figure 14 shows the QSAR model plot of correlations representing the observed versus predicted IC50 μM values for the bio-active training set. The plots are uniformly scattered.

Equation:

IC50 = −1.0859 + 1.294 [Dipole_X] + 0.013735 [Jurs_DPSA_1].

##### Validation of QSAR

**Figure 15:**
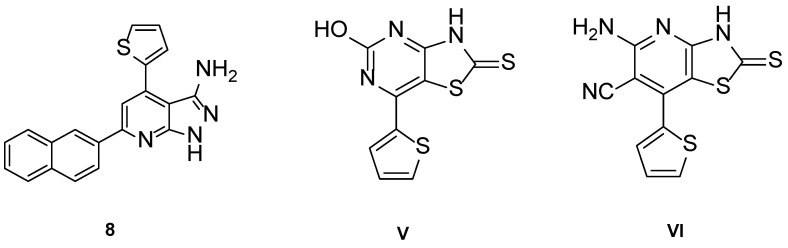
External test set used in validation of generated QSAR model.

Internal and external validation of the determined QSAR equations was performed. The internal validation was presented in Table 4 including determination of r2 (the coefficient of determination), r2 adj (adjusted for the number of terms in the model), r2 pred (the prediction), q2 (from a leave-1-out cross-validation), Friedman L.O.F. (the Friedman lack-of-fit score), and S.O.R. *p*-value (the *p*-value for significance of regression). The external validation was carried out utilizing compound **8** and two reported compounds **V**, **VI** with similar scaffold (Figure 15) [6,48]. The estimated activity for the external test set was very close to its observed activity (Table 5), that is, support the established QSAR model in the present work. In addition, QSAR model_ Applicability showed that all properties and OPS components are within expected ranges, with Applicability MD value 2.66667, and the applicability ranging from 0.236031 to 4.45003 for each component of the training set.

#### 2.2.4. Structure–Activity Relationship (SAR)

Structure–activity relationships among the newly synthesized pyridine derivatives have been closely investigated. The presence of the thione group in compound **7** with thiophene moiety increases antitumor activity against HepG2 and A549. Further investigations of the biological results concluded that the furopyridine derivative as in compound **14** and incorporating the ester functionality showed superiority in activity over the pyridine derivatives against a colon cancer cell line.

## 3. Materials and Methods

### 3.1. Chemistry

All melting points were measured using a Reichert Thermovar apparatus and are uncorrected. Yields listed are of isolated compounds. The IR spectra were recorded on a Perkin-Elmer model 1720 FTIR spectrometer for KBr disc. ^1^H and ^13^C-NMR spectra were recorded on a Bruker AC-300 or DPX-300 spectrometer. Chemical shifts were reported in δ scale (ppm) relative to TMS as a reference standard and the coupling constants J values are given in Hz. Mass spectra were recorded on a GC/MS SHIMADZU spectrophotometer. The progress of the reactions was monitored by TLC using aluminum silica gel plates 60 F245. IR, ^1^H-NMR, ^13^C-NMR, GC-MS, and elemental analyses were performed at the Micro analytical center at the Faculty of science, Cairo University, Cairo, Egypt. Compounds **1** and 13 were prepared according to a previous procedure [44].

#### 3.1.1. 3-Cyano-6-(naphthalen-2-yl)-4-(thiophen-2-yl)pyridin-2-yl 2-chloroacetate (**2**)

To a solution of compound **1** (3.28 g, 10 mmol) and anhydrous potassium carbonate (1.38 g, 10 mmol) in dry acetone (30 mL), chloroacetyl chloride (0.79 mL, 10 mmol) was added drop wise. The reaction mixture was stirred at room temperature for 8 h. The mixture was poured onto cold water and extracted with ethyl acetate, dried over anhydrous sodium sulfate, and concentrated under vacuum to give compound **2**.

Yellow crystals; Yield: 84%; m.p. 165–166 °C; IR (KBr, ν, cm^−1^): 2974 (aliphatic C–H), 2216 (CN), 1697 (C=O), 1596 (C=N); ^1^H-NMR (DMSO-d_6_): δ (ppm) 4.40 (s, 2H, CH_2_Cl), 6.73–7.98 (m, 10H, Ar-H, thiophene-H), 7.85 (s, 1H, pyridine-H); ^13^C-NMR: 40.1 (CH_2_-Cl), 94.0 (pyridine-C^3^), 114.6 (CN), 120.4 (pyridine-C^5^), 126.2–138.2 (Ar-C), 149.7 (pyridine-C^4^), 157.7 (pyridine-C^6^), 161.9 (pyridine-C^2^), 165.4 (C=O). Anal. Calc. for C_22_H_13_ClN_2_O_2_S (404.87): C, 65.27; H, 3.24; N, 6.92, S, 7.92, Cl, 8.76. Found: C, 65.35; H, 3.40; N, 6.90, S, 7.94, Cl, 8.77.

#### 3.1.2. 6-(Naphthalen-2-yl)-4-(thiophen-2-yl)-2-((2-thioxo-2,5-dihydro-1H-imidazol-4-yl)oxy)nicotinonitrile (**3**)

To a solution of compound **2** (4.06 g, 10 mmol) and anhydrous potassium carbonate (1.38 g, 10 mmol) in DMF (20 mL), thiourea (0.76 g, 10 mmol) was added, and the reaction mixture was heated under reflux for 8 h. After cooling, the reaction mixture was poured onto ice cold water. The formed precipitate was collected by filtration and crystallized from methanol to give compound **3**.

Brown crystals; Yield: 88%; m.p. 172–173 °C; IR (KBr, ν, cm^−1^): 3424 (NH), 2214 (CN), 1647 (C=N), 1260 (C=S); ^1^H-NMR (DMSO-d_6_): δ (ppm) 4.13 (s, 2H, imidazole-H), 6.83–8.06 (m, 10H, Ar-H, thiophene-H), 7.82 (s, 1H, pyridine-H), 12.98 (s, 1H, NH, D_2_O exchangeable); ^13^C-NMR: 50.6 (imidazole-C^4^), 98.2 (pyridine-C^3^), 104.6 (pyridine-C^5^), 115.4 (CN), 126.3–139.9 (Ar-C), 145.4 (pyridine-C^4^), 151.1 (pyridine-C^6^), 168.1 (imidazole-C^5^), 170.2 (pyridine-C^2^), 188.4 (C=S). Anal. Calc. for C_23_H_14_N_4_OS_2_ (426.51): C, 64.77; H, 3.31; N, 13.14; S, 15.03. Found: C, 64.67; H, 3.30; N, 13.41; S, 15.00.

#### 3.1.3. 2-Methoxy-6-(naphthalen-2-yl)-4-(thiophen-2-yl)nicotinonitrile (**5**)

A solution of compound **4** (3.46 g, 10 mmol) in methanol (20 mL) and sodium methoxide (0.54 g, 10 mmol) was refluxed for 3 h. After cooling, the reaction mixture was poured onto water, and the solid obtained was crystallized from ethanol to give compound **5**.

Brown crystals; Yield: 75%; m.p. 125–126 °C; IR (KBr, ν, cm^−1^): 2216 (CN), 1652 (C=N); ^1^H-NMR (DMSO-d_6_): δ (ppm) 4.16 (s, 3H, OCH_3_), 7.55–8.06 (m, 10H, Ar-H, thiophene-H), 7.90 (s, 1H, pyridine-H); ^13^C-NMR: 55.5 (OCH_3_), 90.3 (pyridine-C^3^), 110.1 (pyridine-C^5^), 115.5 (CN), 127.4–139.6 (Ar-C), 149.7 (pyridine-C^4^), 156.5 (pyridine-C^6^), 166.2 (pyridine-C^2^). Anal. Calc. for C_21_H_14_N_2_OS (342.42): C, 73.66; H, 4.12; N, 8.18; S, 9.36. Found: C, 73.60; H, 4.32; N, 8.18; S, 9.32.

#### 3.1.4. 6-(Naphthalen-2-yl)-2-(phenylamino)-4-(thiophen-2-yl)nicotinonitrile (**6**)

A mixture of compound **4** (3.46 g, 10 mmol) and aniline (0.9 mL, 10 mmol) in methanol (20 mL) containing a few drops of pyridine was refluxed for 8 h. The solid obtained after cooling was poured on ice/water-containing HCl, filtered off, air dried on suction, and crystallized from acetic acid to give compound **6**.

Yellow crystals; Yield: 84%; m.p. 130–131 °C; IR (KBr, ν, cm^−1^): 3428 (NH), 2365 (CN), 1604 (C=N); ^1^H-NMR (DMSO-d_6_): δ (ppm) 6.83–7.93 (m, 15H, Ar-H, thiophene-H), 7.81 (s, 1H, pyridine-H), 9.80 (s, 1H, NH, D_2_O exchangeable); ^13^C-NMR: 86.3 (pyridine-C^3^), 109.2 (pyridine-C^5^), 114.8 (CN), 111.7–138.3 (Ar-C), 148.9 (pyridine-C^4^), 157.4 (pyridine-C^6^), 164.7 (pyridine-C^2^). Anal. Calc. for C_26_H_17_N_3_S (403.50): C, 77.39; H, 4.25; N, 10.41; S, 7.95. Found: C, 77.45; H, 4.30; N, 10.44; S, 8.00.

#### 3.1.5. 6-(Naphthalen-2-yl)-4-(thiophen-2-yl)-1H-pyrazolo[3,4-b]pyridin-3-amine (**8**)

A solution of compound **7** (3.44 g, 10 mmol) and hydrazine hydrate (0.47 mL, 15 mmole) in absolute ethanol (20 mL) was heated under reflux for 6 h. The reaction mixture was cooled. The solid that precipitated was filtered off and crystallized from ethyl acetate to give compound **8**.

Brown crystals; Yield: 72%; m.p. 175–176 °C; IR (KBr, ν, cm^−1^): 3375 and 3228 (NH and NH_2_), 1629 (C=N); ^1^H-NMR (DMSO-d_6_): δ (ppm) 6.45 (s, 2H, NH_2_, D_2_O exchangeable), 7.55–8.22 (m, 10H, Ar-H), 8.01 (s, 1H, pyridine-H), 11.65 (s, 1H, NH, D_2_O exchangeable); ^13^C-NMR: 90.2 (pyrazole-C^4^), 123.9 (pyridine-C^3^), 124.1–135.7 (Ar-C), 151.3 (pyrazole-C^5^), 152.2 (pyrazole-C^3^), 156.1 (pyridine-C^2^). Anal. Calcd for C_20_H_14_N_4_S (342.42): C, 70.15; H, 4.12; N, 16.36. Found: C, 70.30; H, 4.22; N, 16.34.

#### 3.1.6. General Procedure for Preparing Amino Sugar Derivatives 9 and 10

To a solution of compound **8** (3.44 g, 10 mmol) in ethanol (30 mL) and glacial acetic acid (2 drops), *d*-glucose or *d*-galactose was added in water (1 mL). The reaction mixture was refluxed for 6 h (TLC; methanol/chloroform; 0.5/9.5) and then allowed to cool at room temperature. The excess ethanol was removed under reduced pressure, and the residue was treated with diethyl ether (15 mL). The solid that formed was filtered and crystallized from ethanol to form compounds **9**, **10**.

**3-((β-D-glucopyranosylamino)-6-(naphthalen-2-yl)-4-(thiophen-2-yl)-7,7a-dihydro-1*H*-pyrazolo[3,4-b]pyridine (9).** Brown powder; Yield: 86%; m.p. 285–286 °C; IR (KBr, ν, cm^−1^): 3520–3390 (OH), 3419 (NH), 1592 (C=N); ^1^H-NMR (DMSO-d_6_): δ (ppm) 3.00–3.04 (m, 2H, H-6`,6``), 3.59–3.62 (m, 1H, H-5`), 3.73–3.80 (m, 3H, H-4`, H-3`, OH), 4.45–4.47 (m, 1H, OH), 4.73–4.78 (m, 3H, H-2`, 2OH), 5.85 (d, *J* = 9.6 Hz, 1H, H-1`), 6.80–7.80 (m, 10H, Ar-H), 7.77 (s, 1H, pyridine-H), 10.31, 11.29 (2s, 2H, 2NH, D_2_O exchangeable); ^13^C-NMR: 61.6 (C-6`), 70.7 (C-4`), 75.2 (C-2`), 77.2 (C-3`), 81.5 (C-5`), 90.2 (pyridine-C^3^), 92.6 (C-1`), 121.7 (pyridine-C^5^), 127.5–141.9 (Ar-C), 144.9 (pyridine-C^4^), 151.1 (pyridine-C^2^), 154.0 (pyrazole-C), 155.4 (pyridine-C^6^). Anal. Calc. for C_26_H_24_N_4_O_5_S (504.56): C, 61.89; H, 4.79; N, 11.10. Found: C, 61.94; H, 4.85; N, 11.26.

**3-((β-D-galactopyranosylamino)-6-(naphthalen-2-yl)-4-(thiophen-2-yl)-7,7a-dihydro-1*H*-pyrazolo[3,4-b]pyridine (10).** Yellow powder; Yield: 82%; m.p. 281–282 °C; IR (KBr, ν, cm^−1^): 3510–3385 (OH), 3475 (NH); ^1^H-NMR (DMSO-d_6_): δ (ppm) 3.12–3.13 (m, 2H, H-6`,6``), 3.40–3.42 (m, 1H, H-5`), 3.85–3.89 (m, 3H, H-4`, H-3`, OH), 4.39–4.41 (m, 1H, OH), 4.58–4.88 (m, 3H, H-2`, 2OH), 6.17 (d, *J* = 9.6 Hz, 1H, H-1`), 7.11–8.00 (m, 10H, Ar-H), 7.85 (s, 1H, pyridine-H), 10.45, 11.07 (2s, 2H, 2NH, D_2_O exchangeable). Anal. Calc. for C_26_H_24_N_4_O_5_S (504.56): C, 61.89; H, 4.79; N, 11.10. Found: C, 61.60; H, 5.29; N, 11.0.

#### 3.1.7. S-(3-Cyano-6-(naphthalen-2-yl)-4-(thiophen-2-yl)pyridin-2-yl)2-chloroethanethioate (**11**)

To a solution of compound **7** (3.44 g, 10mmol) and anhydrous potassium carbonate (1.38 g, 10 mmol) in dry acetone (30 mL), chloroacetyl chloride (0.79 mL, 10 mmol) was added drop wise. The reaction mixture was stirred at room temperature for about 8 h. Then, the mixture was poured onto water and extracted with ethyl acetate, dried over anhydrous sodium sulfate, and concentrated under vacuum to give compound **11**.

Black powder; Yield: 89%; m.p. 160–161 °C; IR (KBr, ν, cm^−1^): 2921 (aliphatic C–H), 2209 (CN), 1698 (C=O), 1590 (C=N); ^1^H-NMR (DMSO-d_6_): δ (ppm) 4.30 (s, 2H, CH_2_Cl), 7.34–8.31 (m, 10H, Ar-H, thiophene-H), 8.19 (s,1H, pyridine-H); ^13^C-NMR: 47.0 (CH_2_-Cl), 98.3 (pyridine-C^3^), 112.5 (CN), 120.7 (pyridine-C^5^), 126.1–138.6 (Ar-C), 147.3 (pyridine-C^4^), 159.5 (pyridine-C^6^), 184.2 (pyridine-C^2^), 189.0 (C=O). Anal. Calc. for C_22_H_13_ClN_2_OS_2_ (420.93): C, 62.78; H, 3.11; N, 6.66; S, 15.23. Found: C, 62.65; H, 3.34; N, 6.50; S, 15.22.

#### 3.1.8. S-(3-Cyano-6-(naphthlean-2-yl)-4-(thiophen-2-yl)pyridin-2-yl)-2-azidoethanethioate (**12**)

A mixture of compound **11** (4.22 g, 10 mmol) and sodium azide (0.65 g, 10 mmol) in DMF (20 mL) was stirred at room temperature for 12 h. The reaction mixture was diluted with cold water (10 mL) and extracted with chloroform (3 × 10 mL). The combined organic layer was concentrated to afford compound **12**.

Black crystals; Yield: 75%; m.p. 152–153 °C; IR (KBr, ν, cm^−1^): 2972 (aliphatic C–H), 2366 (N_3_), 2206 (CN), 1676 (C=O); ^1^H-NMR (DMSO-d_6_): δ (ppm) 2.42 (s, 2H, CH_2_N_3_), 7.01–8.06 (m, 10H, Ar-H, thiophene-H), 7.95 (s,1H, pyridine-H); ^13^C-NMR: 65.1 (CN_3_), 101.9 (pyridine-C^5^), 110.4 (pyridine-C^3^), 116.0 (CN), 127.4–135.4 (Ar-C), 147,8 (pyridine-C^4^), 171.0 (pyridine-C^6^), 191.0 (C=O). Anal. Calc. for C_22_H_13_N_5_OS_2_ (427.50): C, 61.81; H, 3.07; N, 16.38; S, 7.74. Found: C, 61.51; H, 3.23; N, 16.40; S, 7.75.

#### 3.1.9. Ethyl-3-amino-6-(naphthalen-2-yl)-4-(thiophen-2-yl)furo[2,3-b]pyridine-2-carboxylate (**14**)

Compound **13** (3.28 g, 10 mmol) was heated under reflux in sodium ethoxide solution (0.02 g atom of sodium in 25 mL of absolute ethanol) for 30 min. After cooling, the solid product was filtered and recrystallized from ethanol to afford compound **14**.

Brown powder; Yield: 60%; m.p. 185–186 °C; IR (KBr, ν, cm^−1^): 3437 (NH_2_), 2921 (aliphatic C–H), 1739 (C=O); ^1^H-NMR (DMSO-d_6_): δ (ppm) 1.19–1.23 (t, *J =* 6.7 Hz, 3H, CH_3_), 4.35–4.38 (q, 2H, CH_2_CH_3_), 5.61 (s, 2H, NH_2_), 6.96–8.52 (m, 10H, Ar-H), 8.06 (s, 1H, pyridine-H); ^13^C-NMR: 13.6 (CH_3_), 58.1 (CH_2_), 105.6 (furan-C^3^), 116.6 (pyridine-C^3^), 121.9 (pyridine-C^5^), 126.4–132.1 (Ar-C), 144.2 (furan-C^2^), 145.1 (pyridine-C^4^), 155.1 (pyridine-C^6^), 159.0 (pyridine-C^2^), 162.0 (C=O). Anal. Calc. for C_24_H_18_N_2_O_3_S (414.48): C, 69.55; H, 4.38; N, 6.76. Found: C, 69.60; H, 4.34; N, 6.90.

#### 3.1.10. 7-(Naphthalen-2-yl)-9-(thiophen-2-yl)pyrido[3′,2′:4,5]furo[3,2-d]pyrimidin-4(3H)-one (**15**)

A mixture of compound **14** (3.28 g, 10 mmol) and formamide (20 mL) was heated under reflux for 5 h. The solid product that formed on cooling was collected and recrystallized from DMF to give compound **15**.

Black powder; Yield: 77%; m.p. 197–198 °C; IR (KBr, ν, cm^−1^): 3419 (NH), 1662 (C=O); ^1^H-NMR (DMSO-d_6_): δ (ppm) 7.24–8.11 (m, 10H, Ar-H), 8.26 (s, 1H, pyridine-H), 8.34 (s, 1H, pyrimidine-H), 11.18 (s, 1H, NH); ^13^C-NMR: 103.4 (furan-C^3^), 116.2 (pyridine-C^3^), 122.7 (pyridine-C^5^), 126.0–140.9 (Ar-C), 144,4 (pyridine-C^4^), 146.1 (pyrimidine-H), 147.8 (furan-C^2^), 157.0 (pyridine-C^6^), 158.5 (pyridine-C^2^), 160.8 (C=O). Anal. Calc. for C_23_H_13_N_3_O_2_S (395.44): C, 69.86; H, 3.31; N, 10.63. Found: C, 69.67; H, 3.51; N, 10.70.

### 3.2. Anti-Cancer Activity

#### 3.2.1. Cell Culture and Maintenance

Dulbecco’s modified Eagle’s medium (DMEM) and fetal bovine serum (FBS) were purchased from Gibco, Paisley, UK. Dimethyl sulfoxide (DMSO) was of HPLC grade, and all other reagents and chemicals were of analytical reagent grade.

Cell cultures of HCT-116 (human colorectal carcinoma), MCF-7 (human breast adenocarcinoma), HepG2 (human hepatocellular carcinoma), and A549 (human lung carcinoma) cell lines were purchased from the American Type Culture Collection (Rockville, MD, USA) and maintained in DMEM medium that was supplemented with 10% heat-inactivated FBS (fetal bovine serum), 100 U/mL penicillin, and 100 U/mL streptomycin. The cells were grown at 37 °C in a humidified atmosphere of 5% CO_2_.

#### 3.2.2. Cytotoxicity Measurement

The anticancer activity against HCT-116, HepG2, A549, and MCF-7 human cancer cell lines was estimated using the 3-[4,5-dimethyl-2-thiazolyl)-2,5-diphenyl-2H-tetrazolium bromide (MTT) assay, which is based on the reduction of the tetrazolium salt by mitochondrial dehydrogenases in viable cells [49,50,51,52]. Cells were dispensed in a 96-well sterile microplate (5 × 10^4^ cells/well) and incubated at 37 °C with a series of different concentrations, in DMSO, of each tested compound or doxorubicin (positive control) for 48 h in a serum-free medium prior to the MTT assay. After incubation, media were carefully removed, and 40 µL of MTT (2.5 mg/mL) were added to each well and then incubated for an additional 4 h. The purple formazan dye crystals were solubilized by the addition of 200 µL of DMSO. The absorbance was measured at 570 nm using a Spectra Max Paradigm Multi-Mode microplate reader. The relative cell viability was expressed as the mean percentage of viable cells compared to the untreated control cells. All experiments were conducted in triplicate and repeated on three different days. All the values were represented as mean ± SD. IC_50_s were determined by probit analysis by SPSS Incprobit analysis (IBM Corp., Armonk, NY, USA).

### 3.3. In Silico Studies

#### Molecular Docking Study

Molecular modeling simulation study was performed through docking of the target compounds in the binding site of CDK2 enzyme using C-Docker protocol in Discovery Studio 4.0 Software. The X-ray crystal structure of Roscovitine in complex with CDK2 was downloaded from http://www.rscb.org/pdb (accessed on 9 March 2021) (PDB code 2A4L) in PDB format. Computational docking is an automated computer-based algorithm designed to estimate two main terms [53]. The first is to determine the suitable position and the orientation of a certain test set molecule’s pose inside the binding site in comparison to that of the X-ray crystallographic enzyme–substrate complex. The second term is the calculation of the estimated protein ligand interaction energy, which is known as binding energy (docking scoring).

## 4. Conclusions

A new series of substituted pyridine derivatives and its *N*-glycosides (**1**–**5**, **7**, **8**, **11**, **13**, and **14**) were designed and synthesized. All compounds exhibited potent CDK2/cyclin A2 inhibitory activity with IC_50_ values ranging 3.52–0.24 μM compared to roscovitine (IC_50_ 0.394 ± 0.0 μM). Amongst them, compound **4** revealed the highest IC_50_ of 0.236 μM. Most of the new compounds exhibited potent antiproliferative activity against HCT-116, MCF-7, HepG2, and A549 compared to doxorubicin. The results revealed agreement between both the experimental and estimated data through docking. The most active compounds **1**, **4**, **8**, **11**, and **14** showed high docking scores and low binding energy values, and their binding mode was compatible with the reported binding mode of the reference compound.

## Figures and Tables

**Table 1:** The IC_50_ results of the target compounds against CDK2 enzyme.

Entry	Name	Results CDK2 IC50 (µM) ± SD
**1**	**1**	0.57 ± 0.1
**2**	**2**	1.789 ± 0.1
**3**	**3**	1.008 ± 0.1
**4**	**4**	0.236 ± 0.1
**5**	**5**	3.527 ± 0.2
**6**	**7**	1.268 ± 0.1
**7**	**8**	0.651 ± 0.1
**8**	**11**	0.504 ± 0.1
**9**	**13**	4.45 ± 0.2
**10**	**14**	0.928 ± 0.1
**11**	Roscovitine	0.394 ± 0.1

**Table 2:** The cytotoxic activities of the compounds against the four cancer cell lines using the MTT assay.

Compound Code	IC_50_ (µM) ± SD			
	HCT-116	MCF-7	HepG2	A549
**1**	39.1 ± 4.2	47.0 ± 4.5	27.9 ± 3.1	60.6 ± 4.8
**2**	43.5 ± 4.5	21.3 ± 3.1	29.4 ± 3.5	70.7 ± 4.1
**3**	49.0 ± 5.1	37.3 ± 4.1	44.8 ± 4.1	57.4 ± 4.5
**4**	45.9 ± 4.1	46.2 ± 4.1	42.9 ± 3.9	67.2 ± 5.1
**5**	33.8 ± 3.5	55.5 ± 4.5	27.2 ± 3.1	47.6 ± 4.5
**7**	37.8 ± 3.9	27.4 ± 2.4	22.7 ± 2.9	36.8 ± 4.1
**8**	33.7 ± 3.3	19.3 ± 2.1	29.1 ± 3.5	43.8 ± 4.1
**11**	31.7 ± 3.5	46.4 ± 4.3	27.6 ± 3.3	56.9 ± 4.6
**13**	32.4 ± 3.1	37.6 ± 3.5	30.2 ± 3.7	45.9 ± 4.3
**14**	31.3 ± 3.3	26.1 ± 3.9	27.9 ± 3.5	56.5 ± 4.8
Doxorubicin	40.0 ± 3.9	64.8 ± 4.1	24.7 ± 3.2	58.1 ± 4.1

**Table 3:** Binding energies and distance of docked compounds into the CDK2 active site.

Compounds NO.	Binding Energy (K.cal/mol^−1^)	Hydrogen Bonding Interaction with the Key Amino Acids	Distance A°
**1**	−51.95	1 HBD through NH group with Leu83	2.84
		1 HBA through an oxygen atom of the carbonyl group with Leu83	2.51
**2**	−42.93	1HBA through an oxygen atom of C=O group with Lys89	2.47
		1HBA through nitrogen atom of CN group with Leu83	2.91
**3**	−44.24	1HBA through sulfur atom of thiophene ring with Leu 83	2.79
**4**	−54.33	2HBA through nitrogen atom of CN group with Leu83	2.53
			2.67
**5**	−41.36	1HBA through nitrogen atom of pyridine with Lys89	3.29
		1HBA through nitrogen atom of CN group with Leu83	3.45
**7**	−46.25	1 HBD through NH group with Leu83	2.84
		2 HBA through sulfur atom of thione and CN group with Asp89	2.69
**8**	−50.9	1HBD through hydrogen atom of NH group of pyrazole ring with Leu83	2.38
		1HBA through nitrogen atom of pyrazole ring with Leu83	2.80
		1HBD through hydrogen atom of NH2 group with Glu81	2.85
**11**	−53.17	1HBA through nitrogen atom of CN group with Leu83	2.54
		2HBA 1HBA through an oxygen atom C=O group with Asp86	2.87, 2.95
**13**	−41.07	1HBA through nitrogen atom of CN group with Leu83	2.94
			3.03
**14**	−49.69	1 HBD through NH2 group with Leu83	2.84
		1HBA through oxygen atom of C=O group with Leu83	2.87
Roscovitine	−55.75	1HBD through hydrogen atom of NH group with Leu83	2.33
		1HBA through nitrogen atom of Imidazole ring with Leu83	2.87

**Table 4:** The internal validation values for the generated 2D-QSAR equation.

Model	r^2^	r^2^ (Adj)	r^2^ (Pred)	RMS Residual Error	Friedman L.O.F.	S.O.R. *p*-Value
IC_50_ = −1.0859 + 1.294 [Dipole_X] + 0.013735 [Jurs_DPSA_1]	0.9179	0.8905	0.8121	0.4811	0.9889	0.0005543

**Table 5:** External validation for the established QSAR models.

Compound No.	Experimental Activity (IC50/μM)	Predicted Activity (IC50/μM)
**8**	0.651	1.020
**V**	0.743	0.947
**VI**	0.139	0.425

## Data Availability

The data presented in this study are available in this article.

## Supplementary Materials

The following are available online, Spectroscopic data of synthesized compounds.

## Sample Availability

Samples of the compounds are available from the authors.
