# Supplementary material for: Discovery of New Pyrazolopyridine, Furopyridine, and Pyridine Derivatives as CDK2 Inhibitors: Design, Synthesis, Docking Studies, and Anti-Proliferative Activity
Source: Molecules. 2021 Jun 26;26(13):3923. doi: 10.3390/molecules26133923 (PMC8272136; doi:10.3390/molecules26133923)
Supplement: Supplementary file 1 [file molecules-26-03923-s001.zip › molecules-1232663-supplementary.pdf]

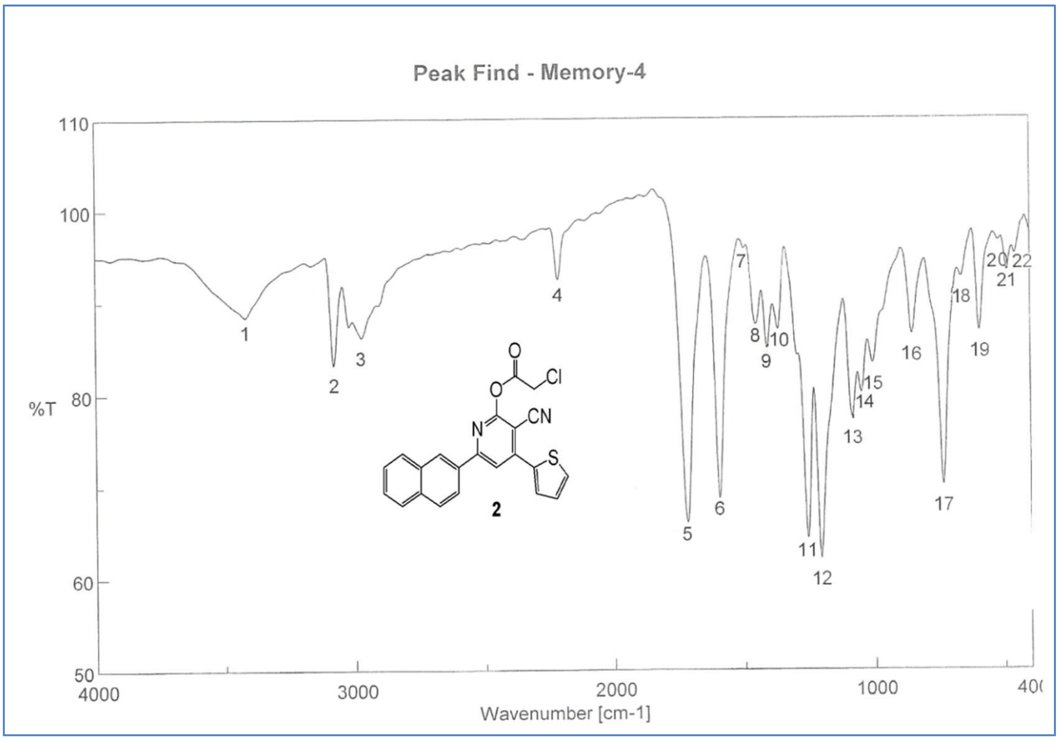

IR spectrum of compound **2**

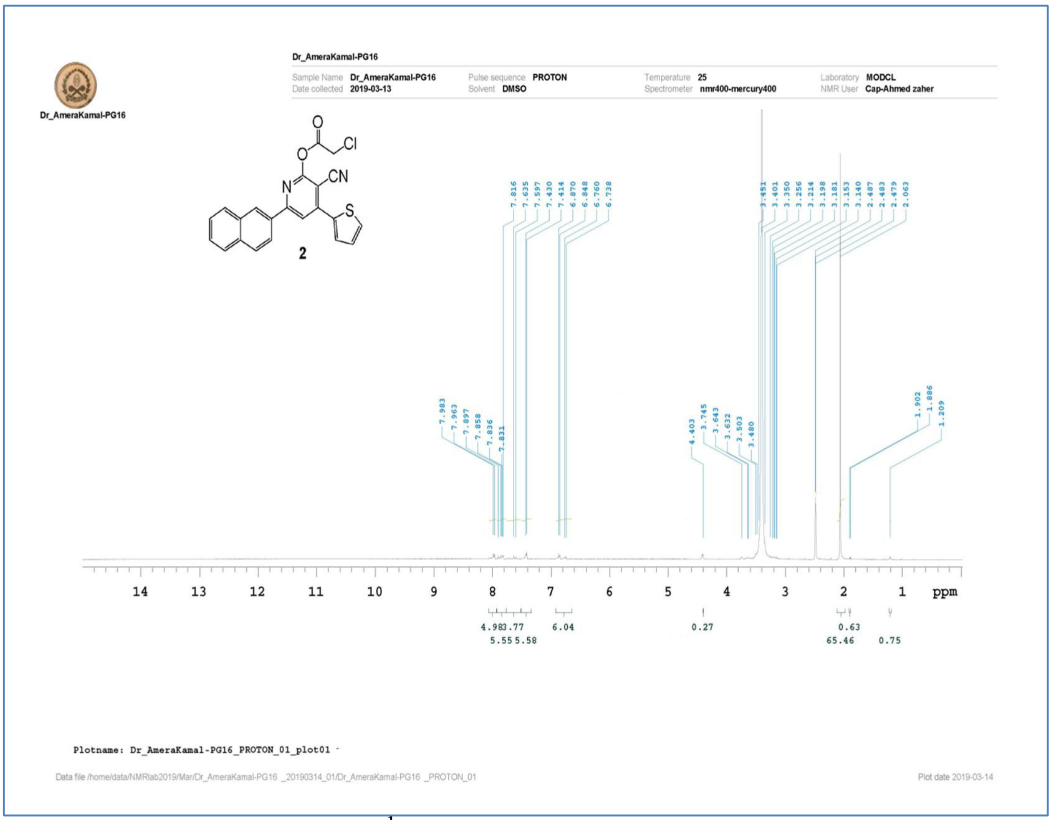

^{1}H NMR of compound **2**

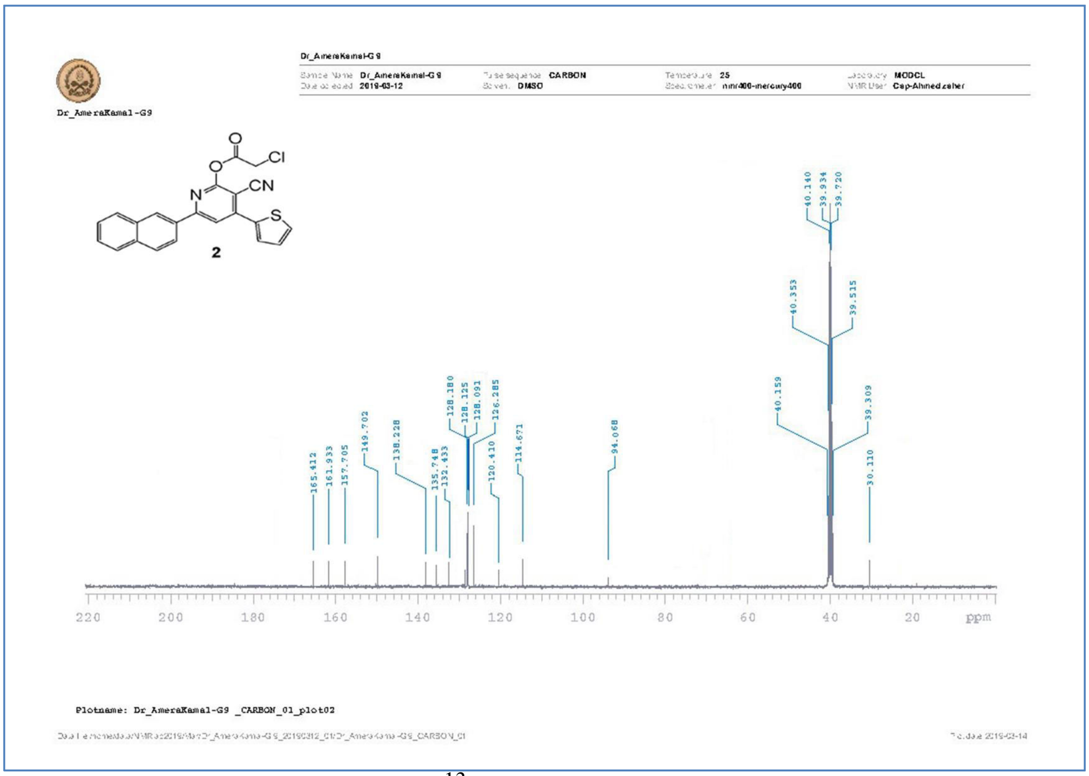

^{13}C NMR of compound **2**

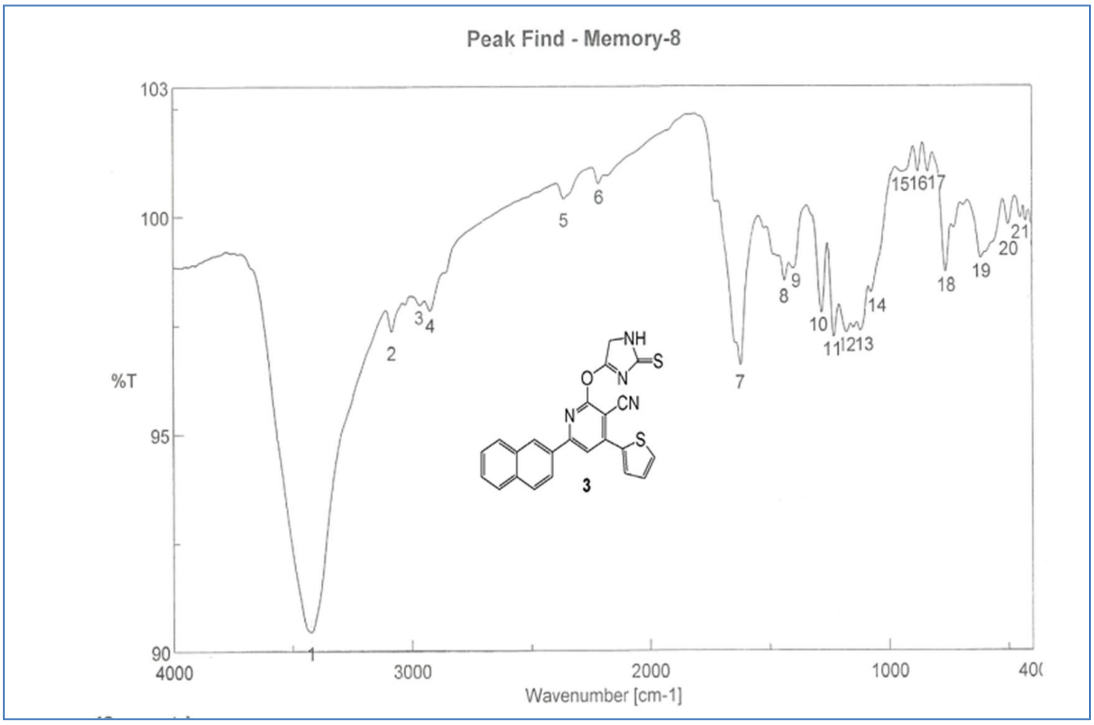

IR spectrum of compound **3**

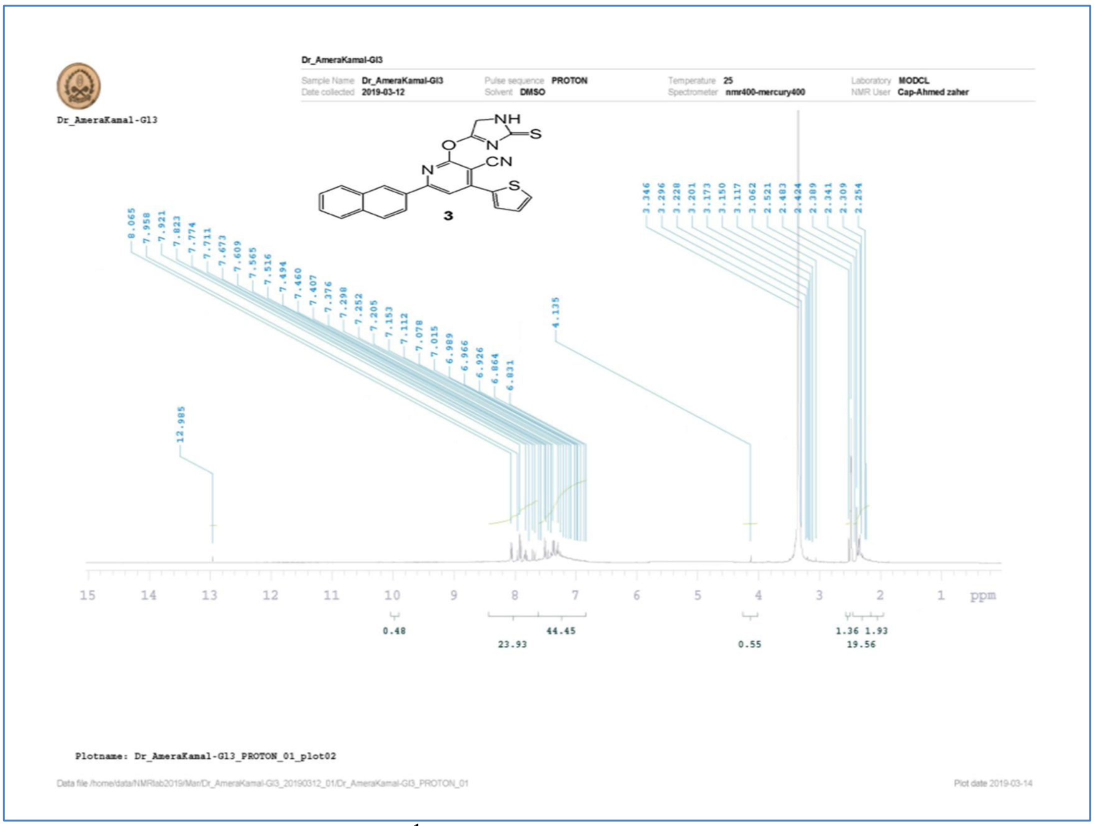

^{1}H NMR of compound **3**

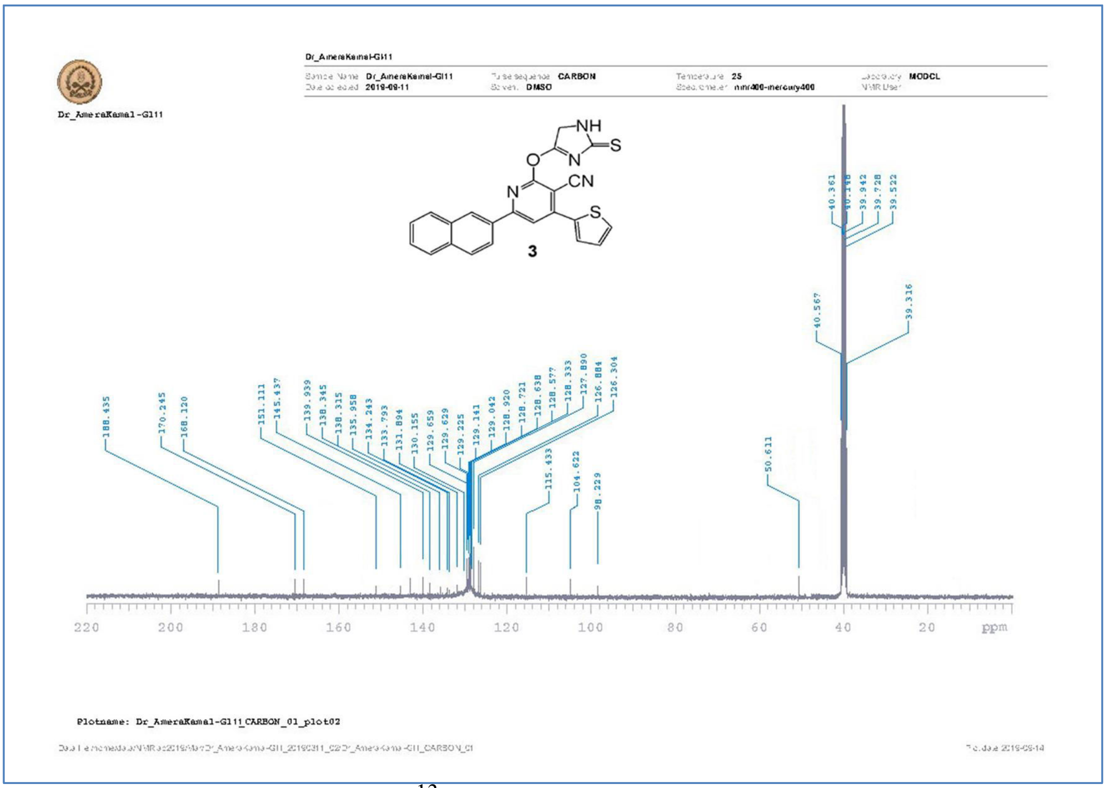

^{13}C NMR of compound **3**

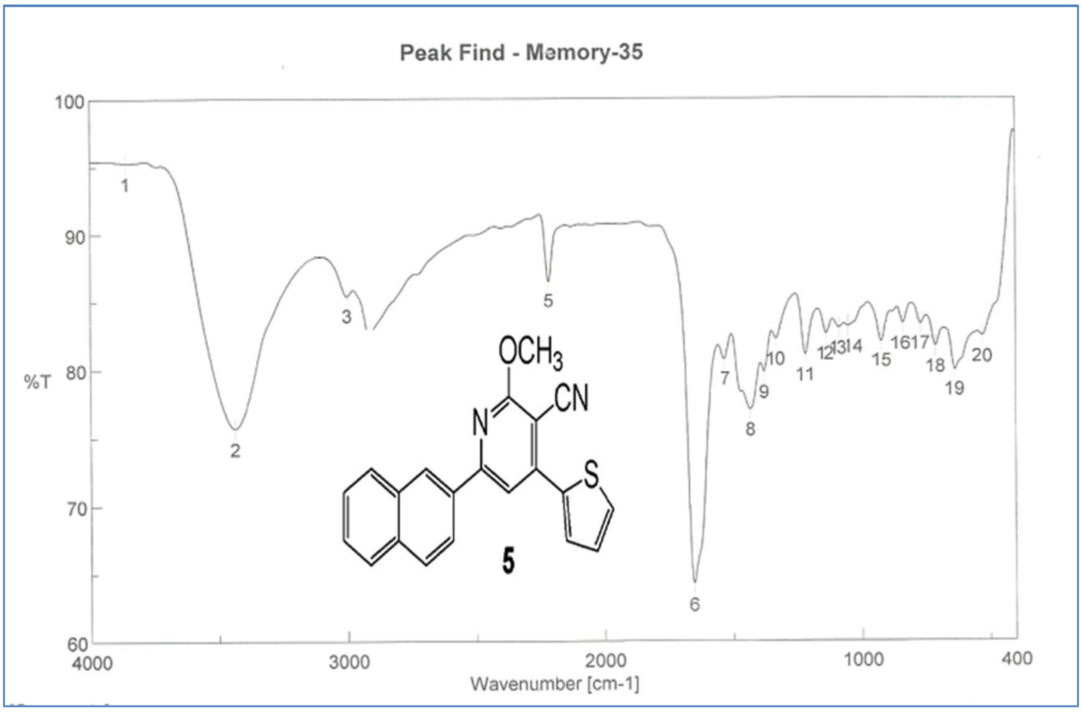

IR spectrum of compound **5**

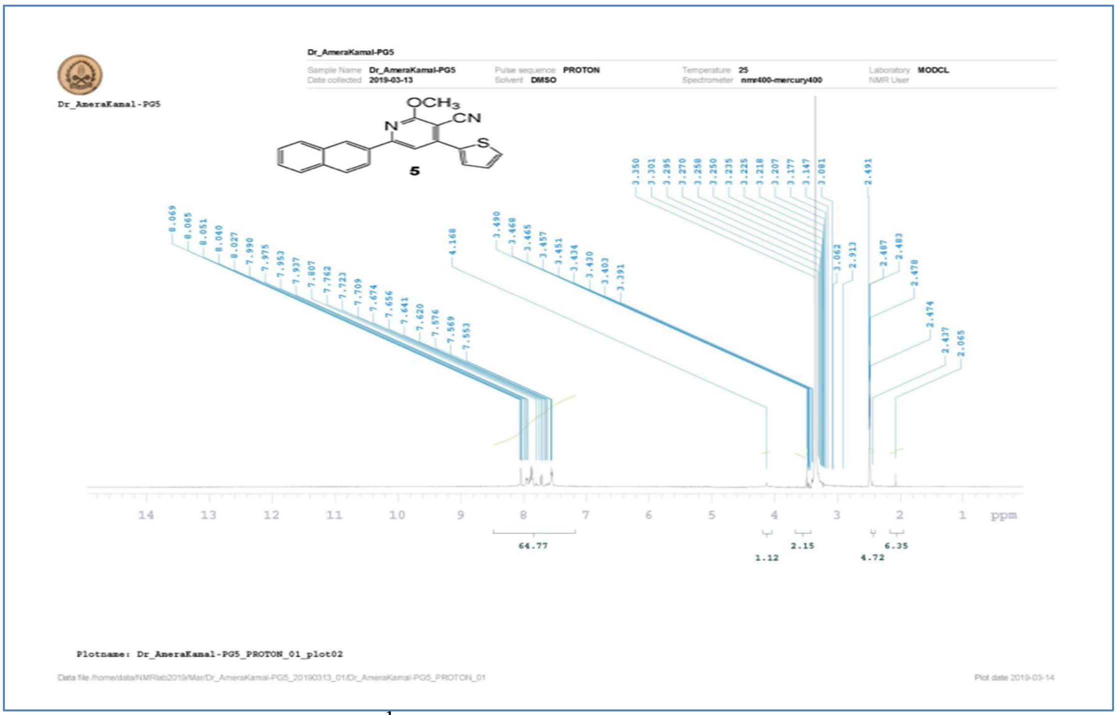

^{1}H NMR of compound **5**

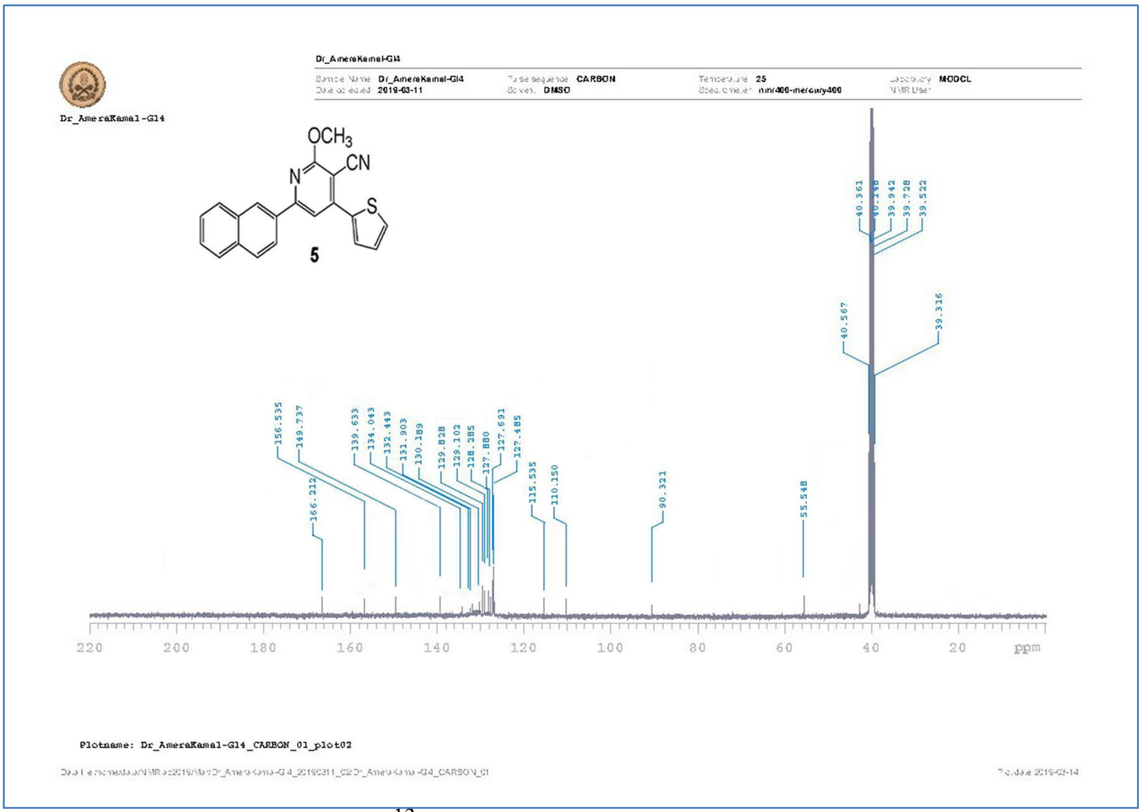

^{13}C NMR of compound **5**

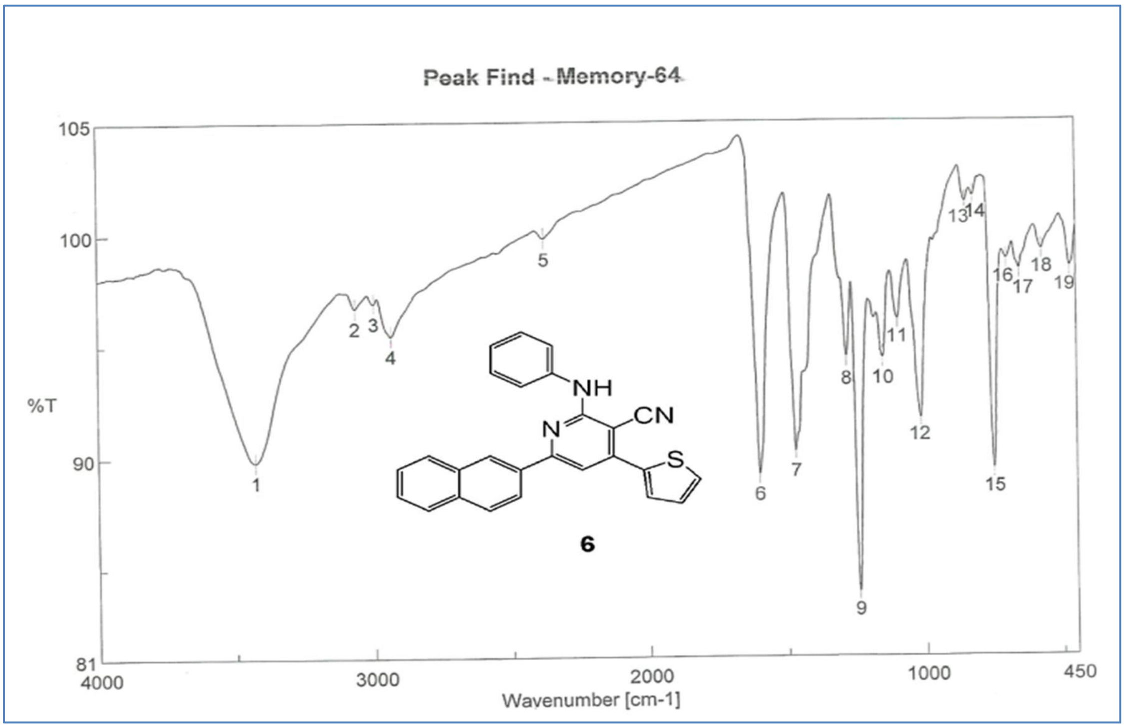

IR spectrum of compound **6**

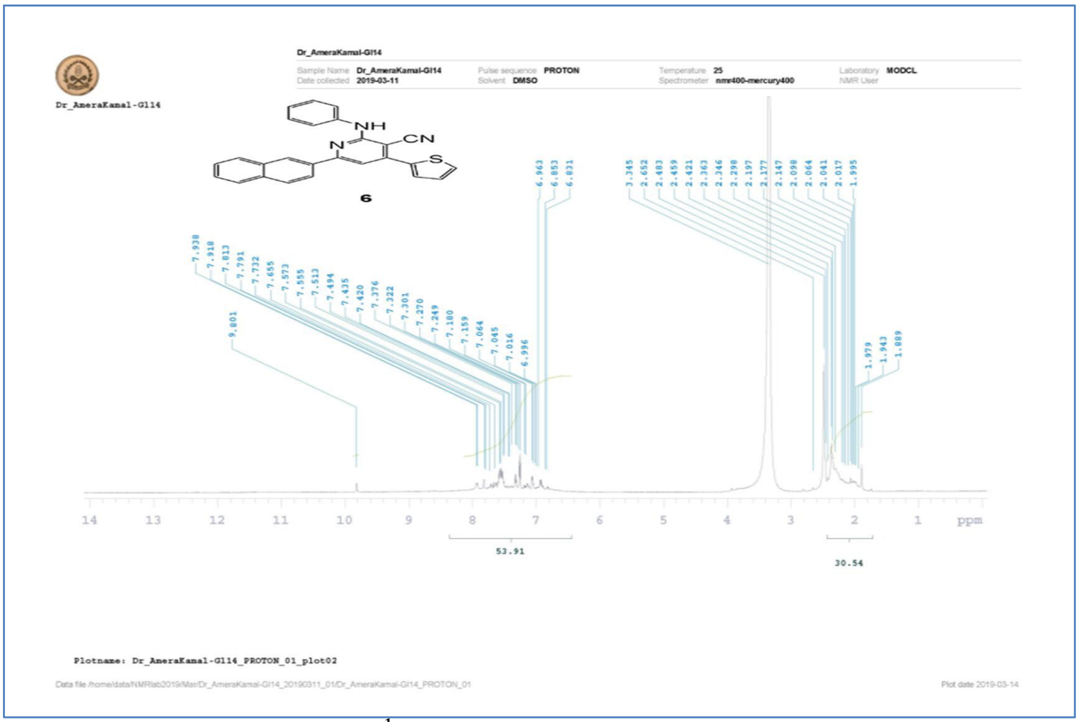

^{1}H NMR of compound **6**

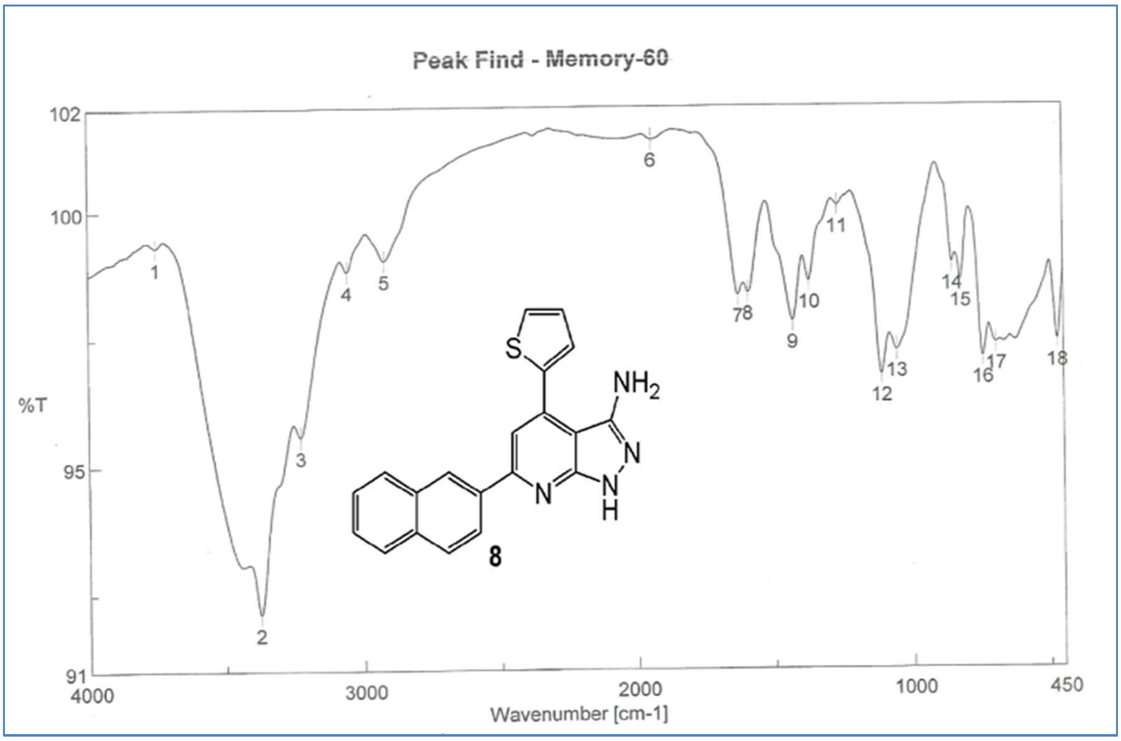

IR spectrum of compound **8**

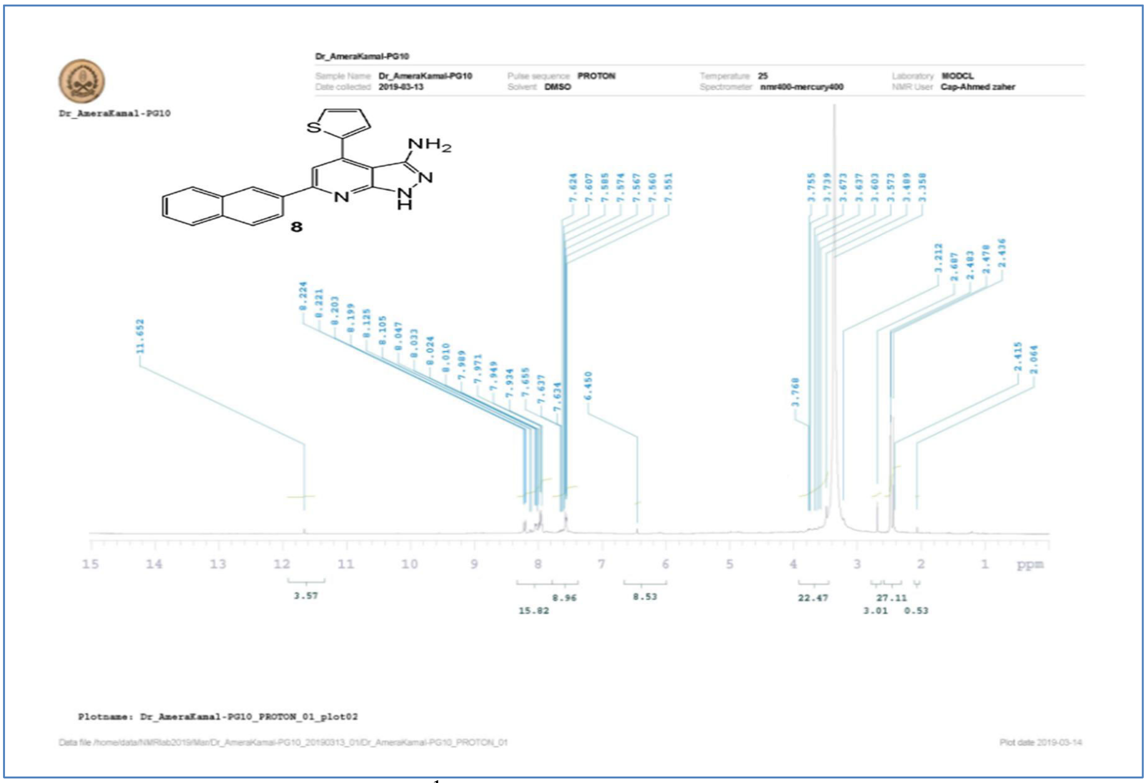

^{1}H NMR of compound **8**

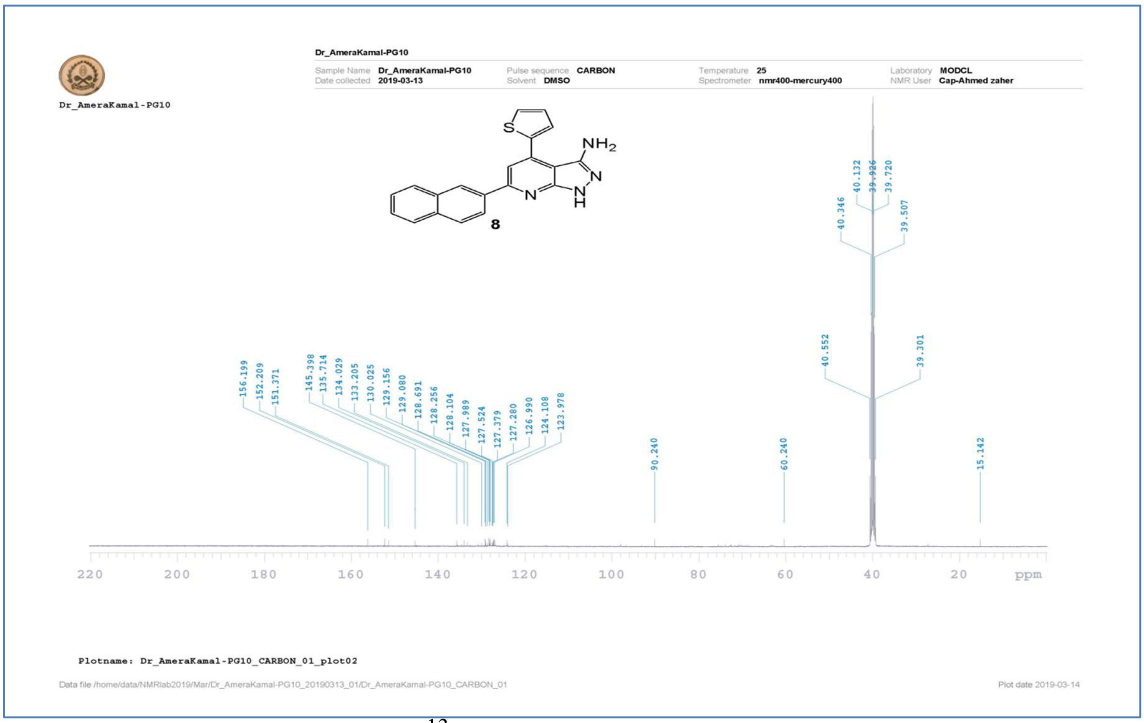

^{13}C NMR of compound **8**

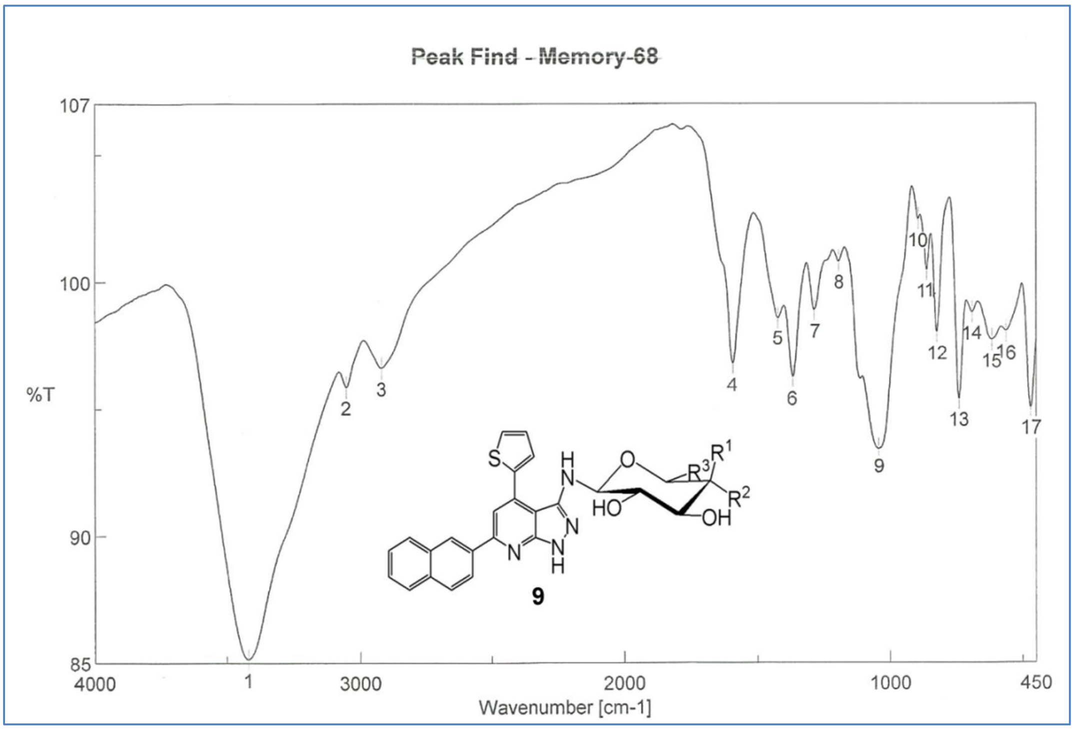

IR spectrum of compound **9**

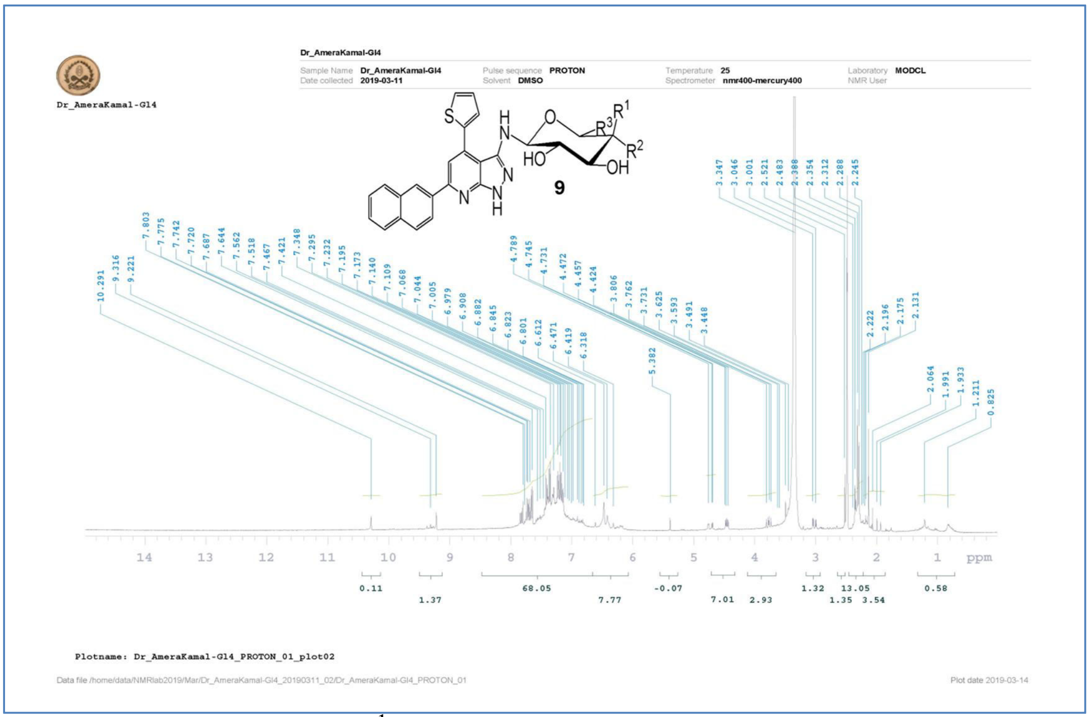

^{1}H NMR of compound **9**

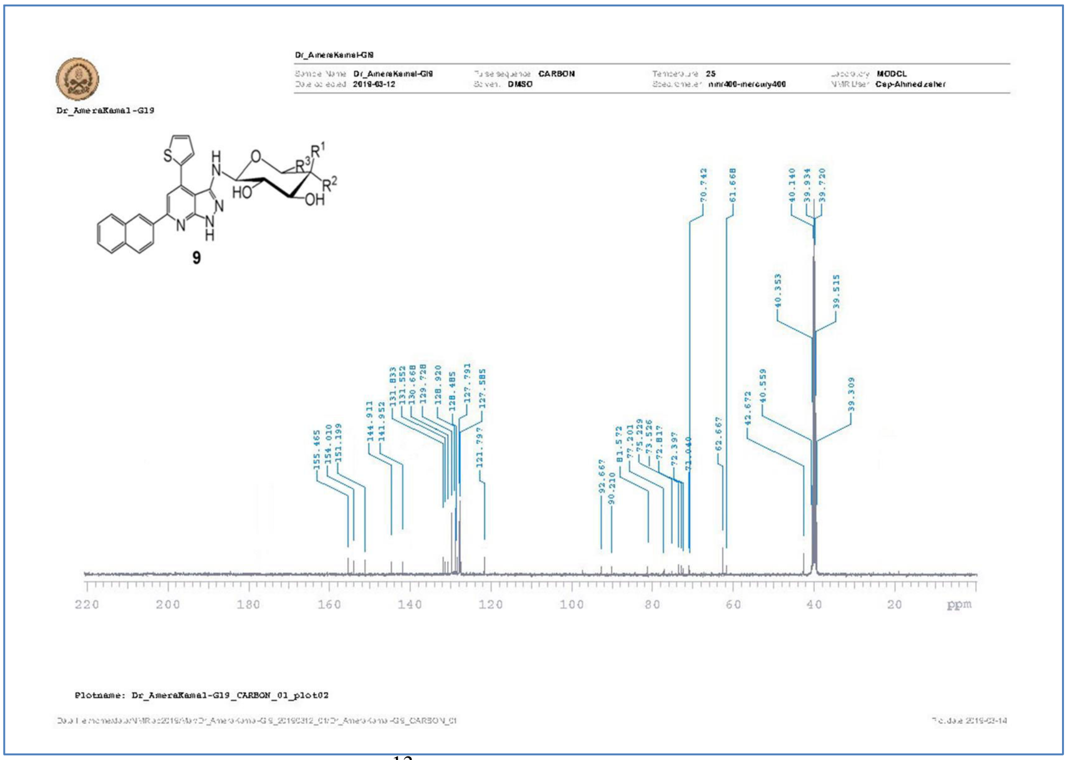

^{13}C NMR of compound **9**

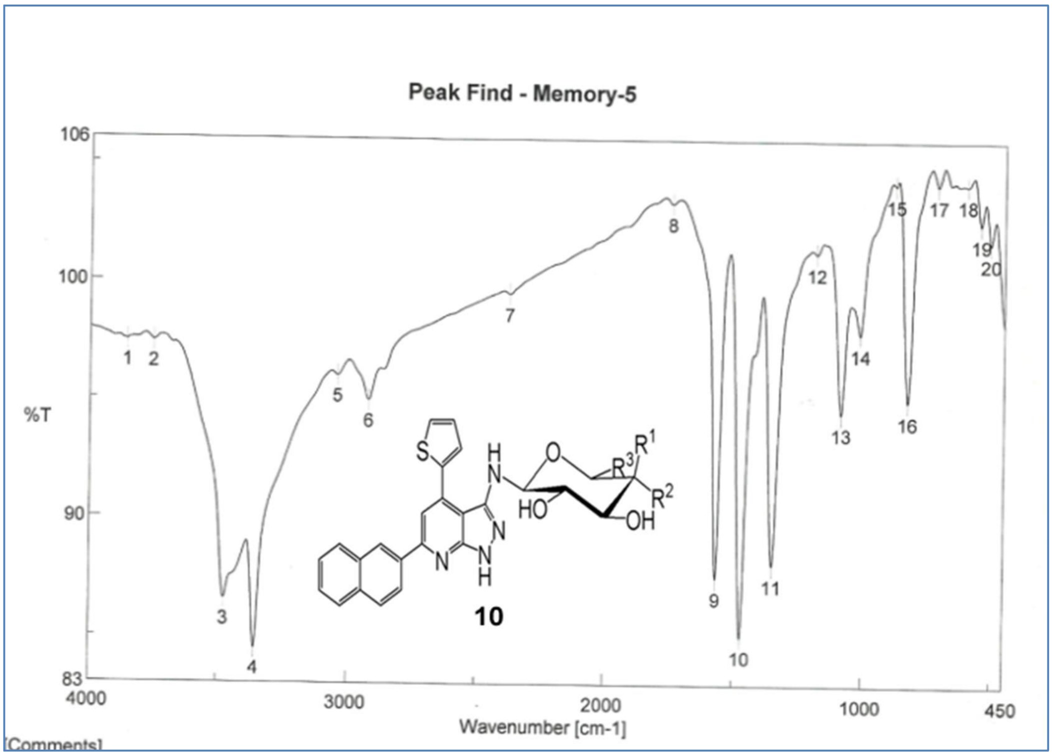

IR spectrum of compound **10**

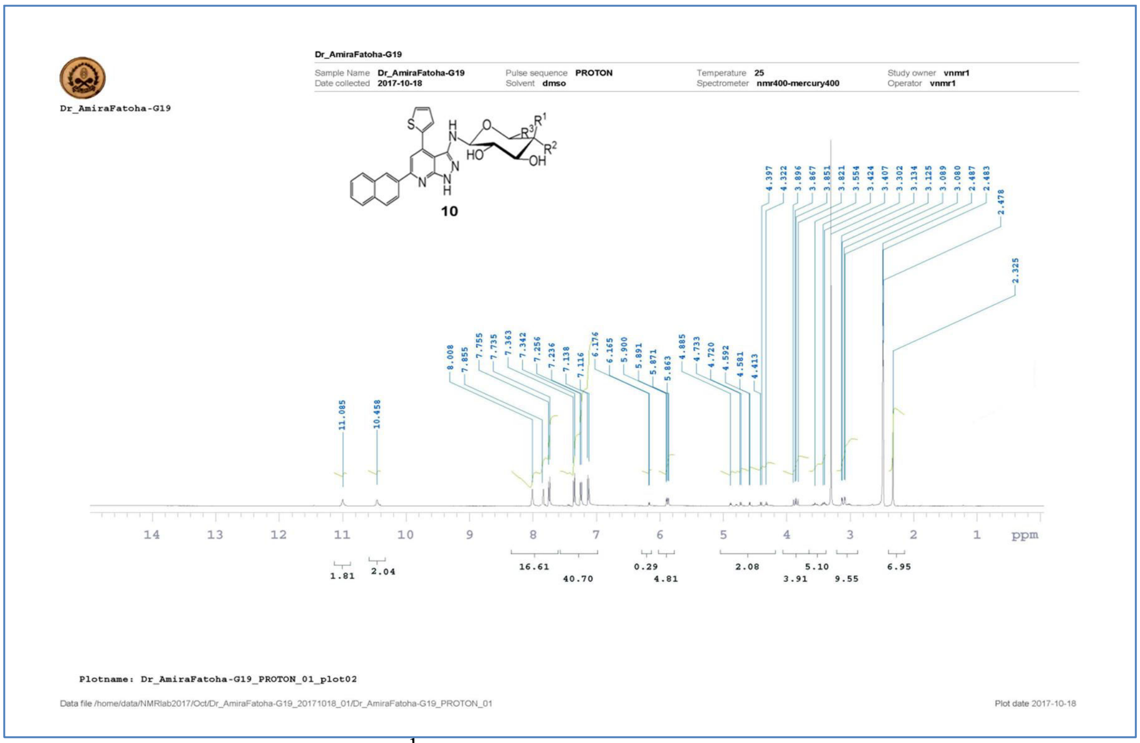

^{1}H NMR of compound **10**

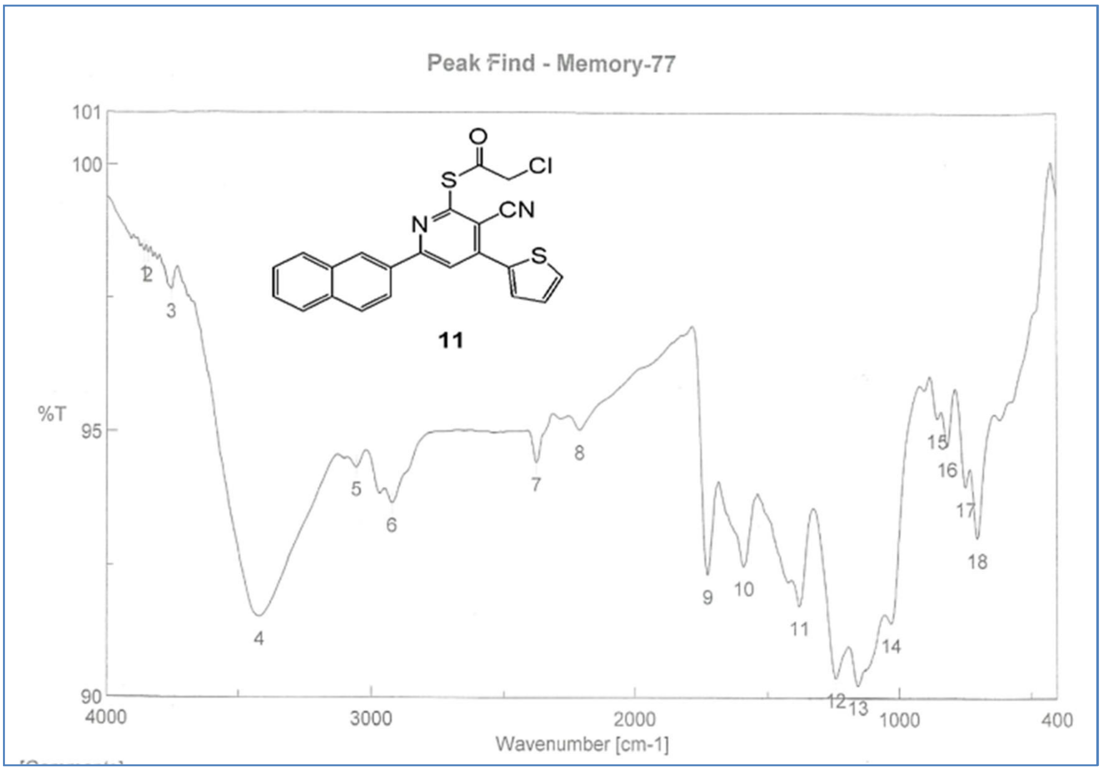

IR spectrum of compound **11**

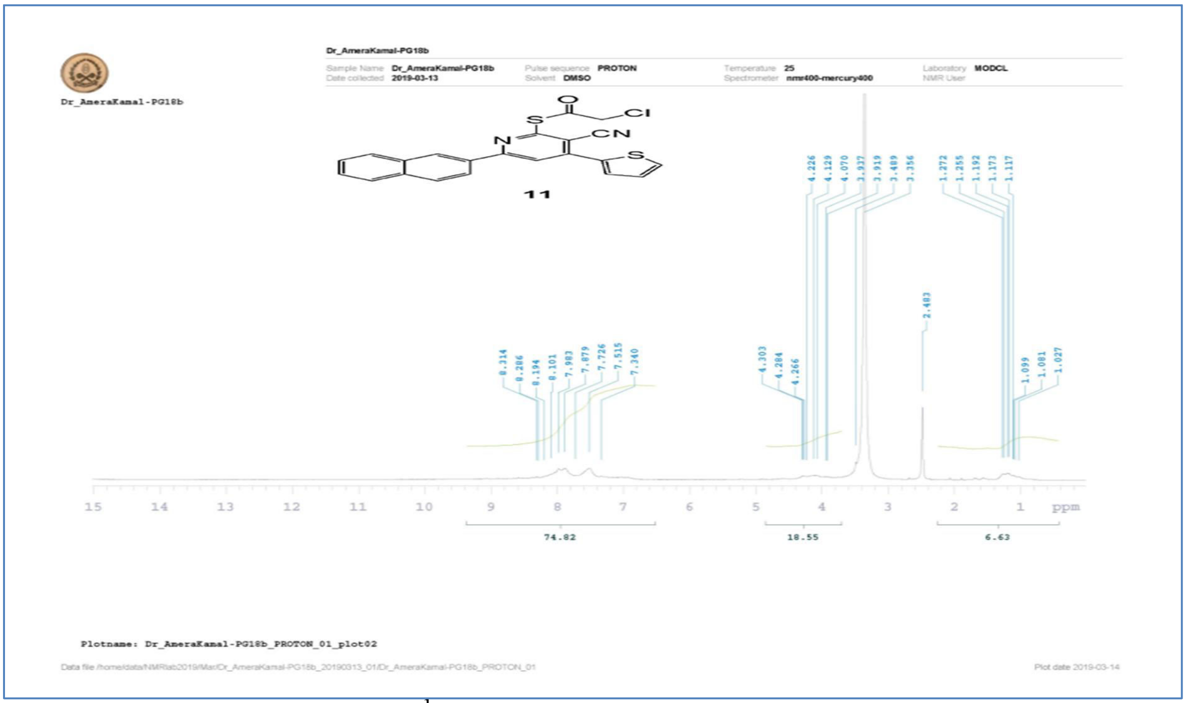

^{1}H NMR of compound **11**

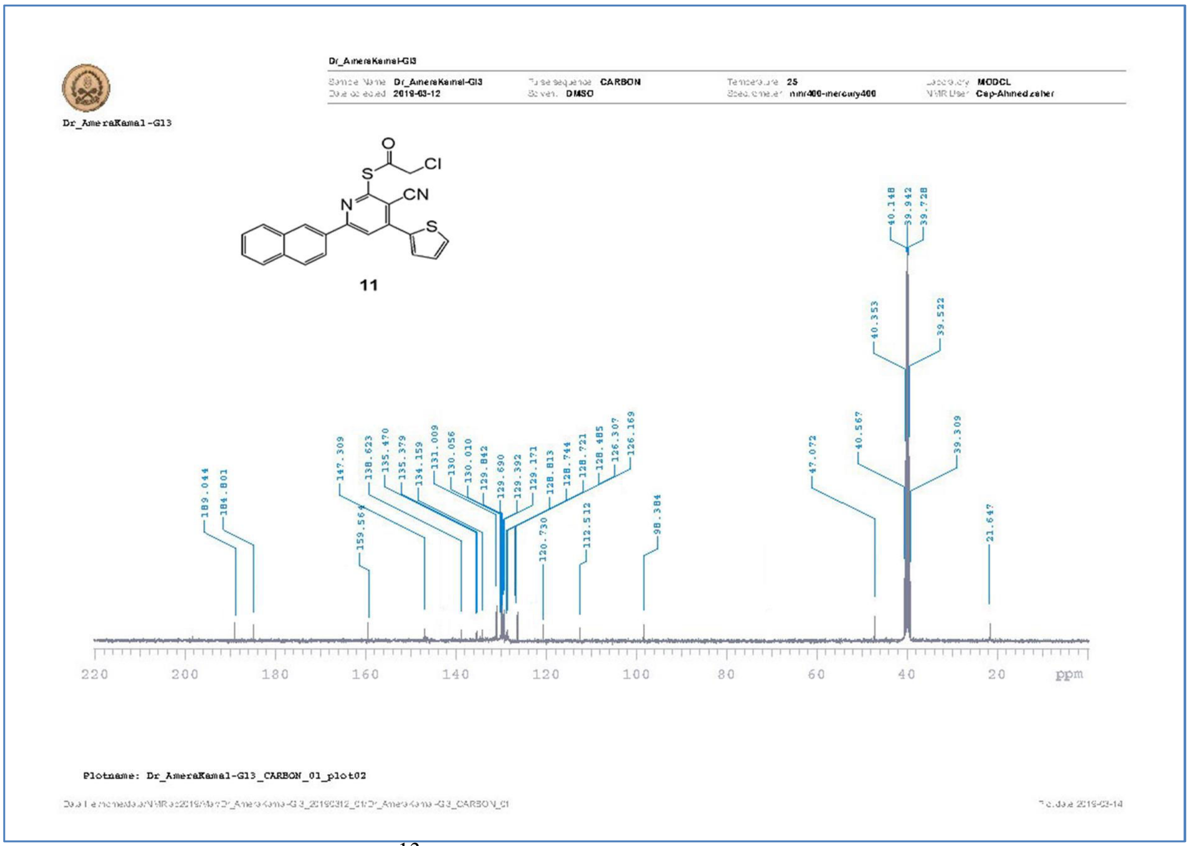

^{13}C NMR of compound **11**

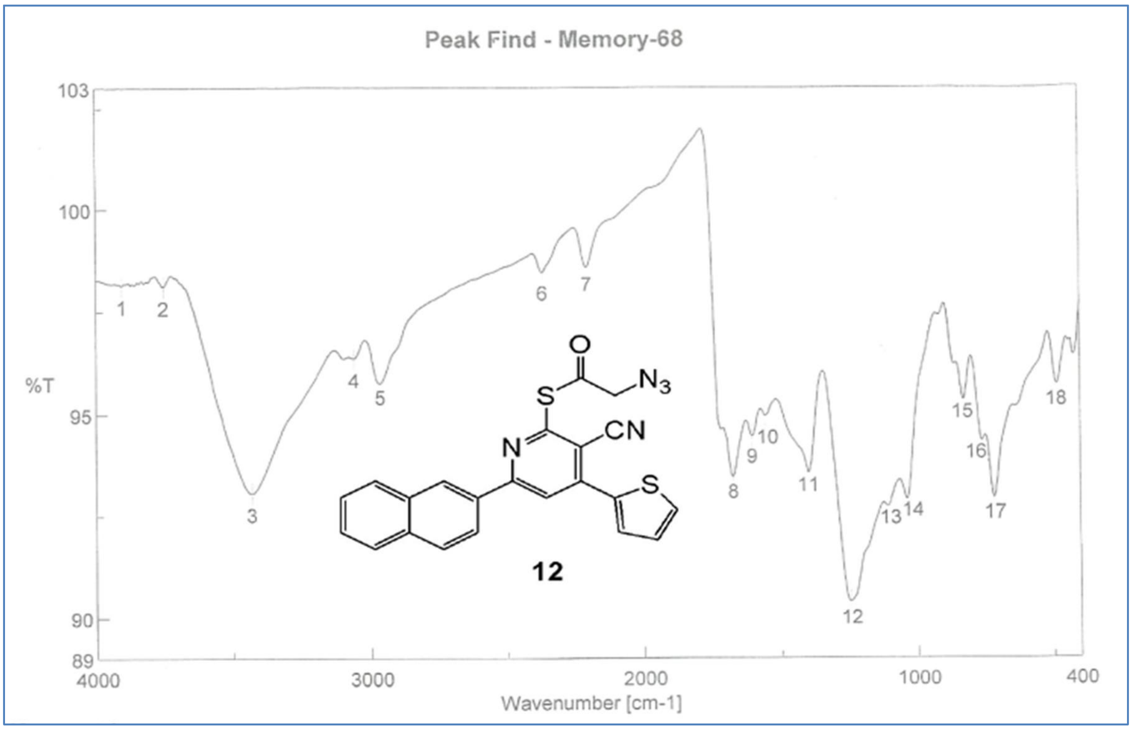

IR spectrum of compound **12**

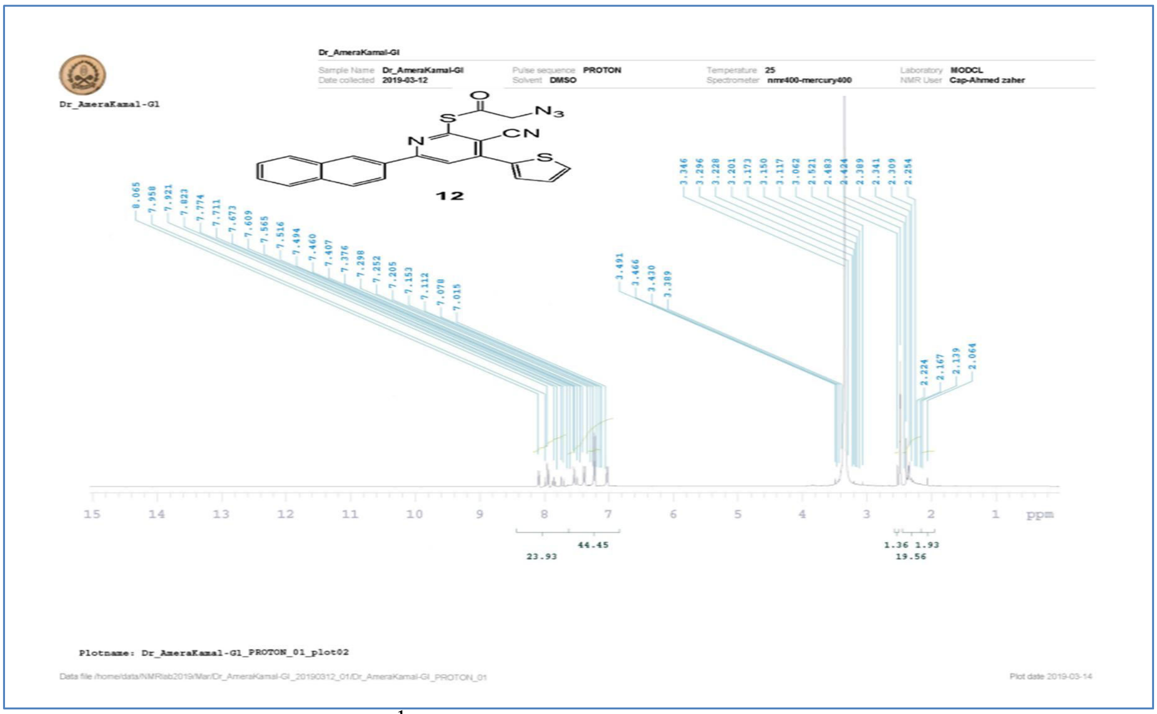

^{1}H NMR of compound **12**

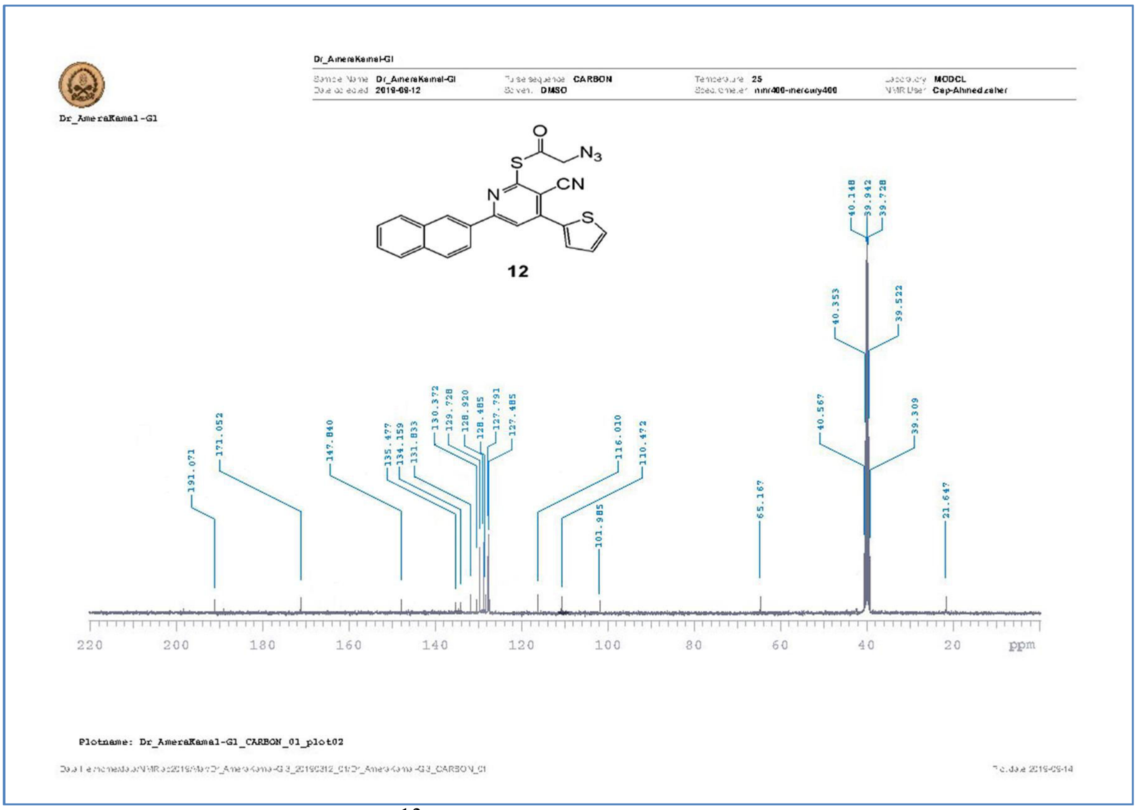

^{13}C NMR of compound **12**

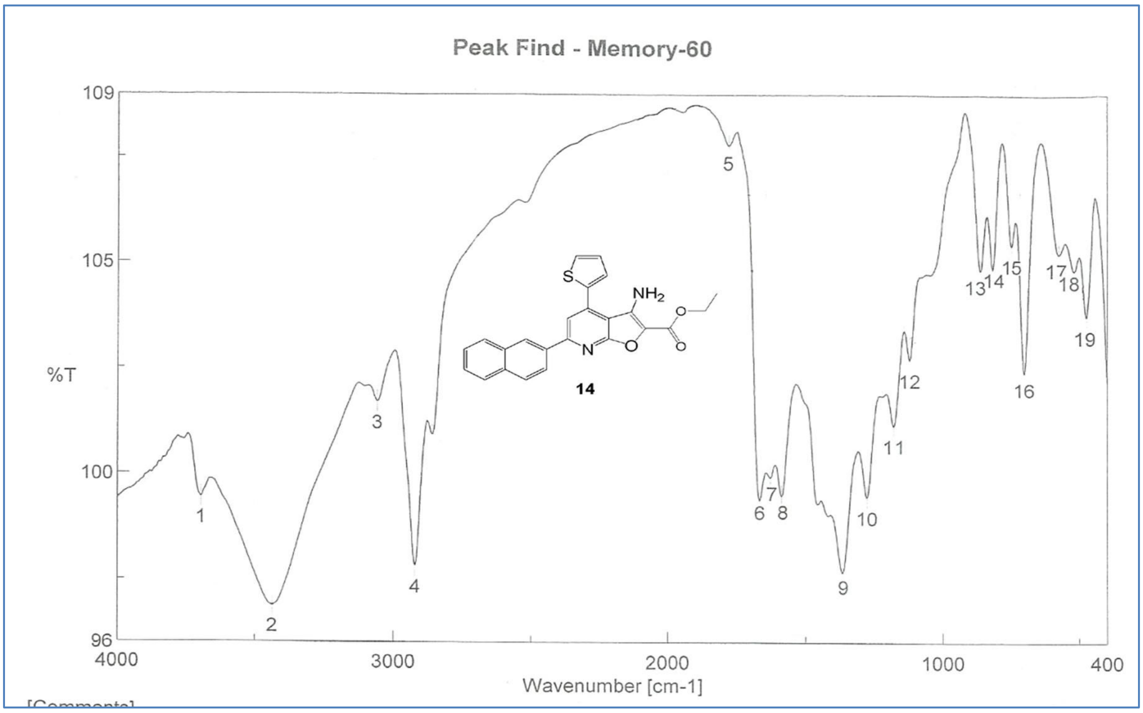

IR spectrum of compound **14**

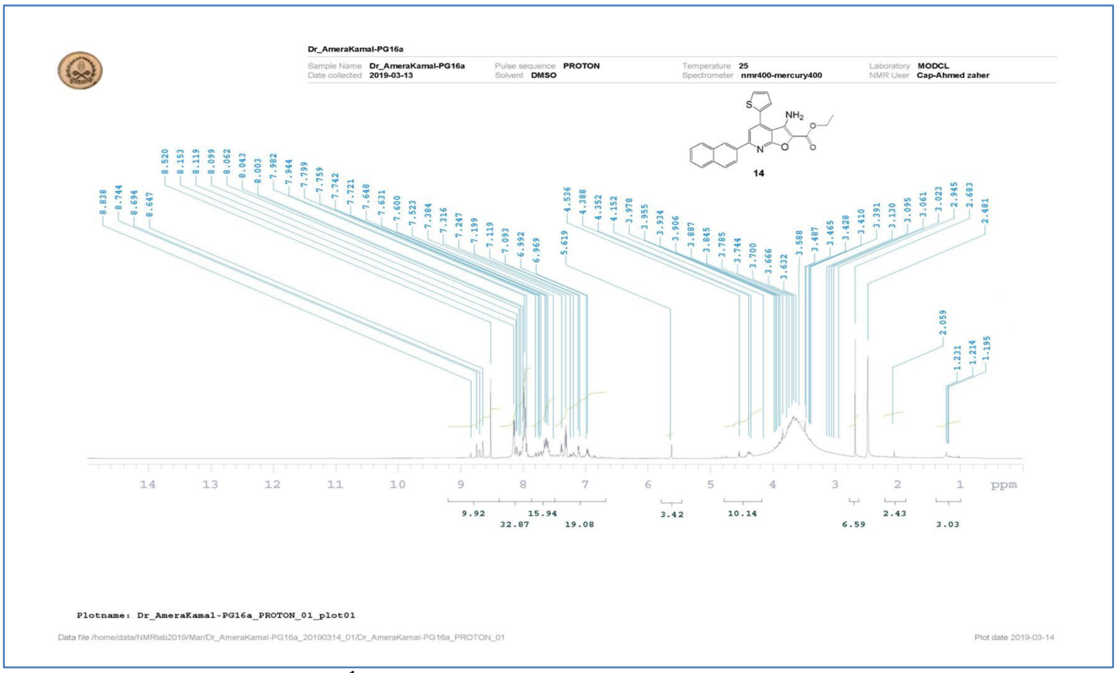

^{1}H NMR of compound **14**

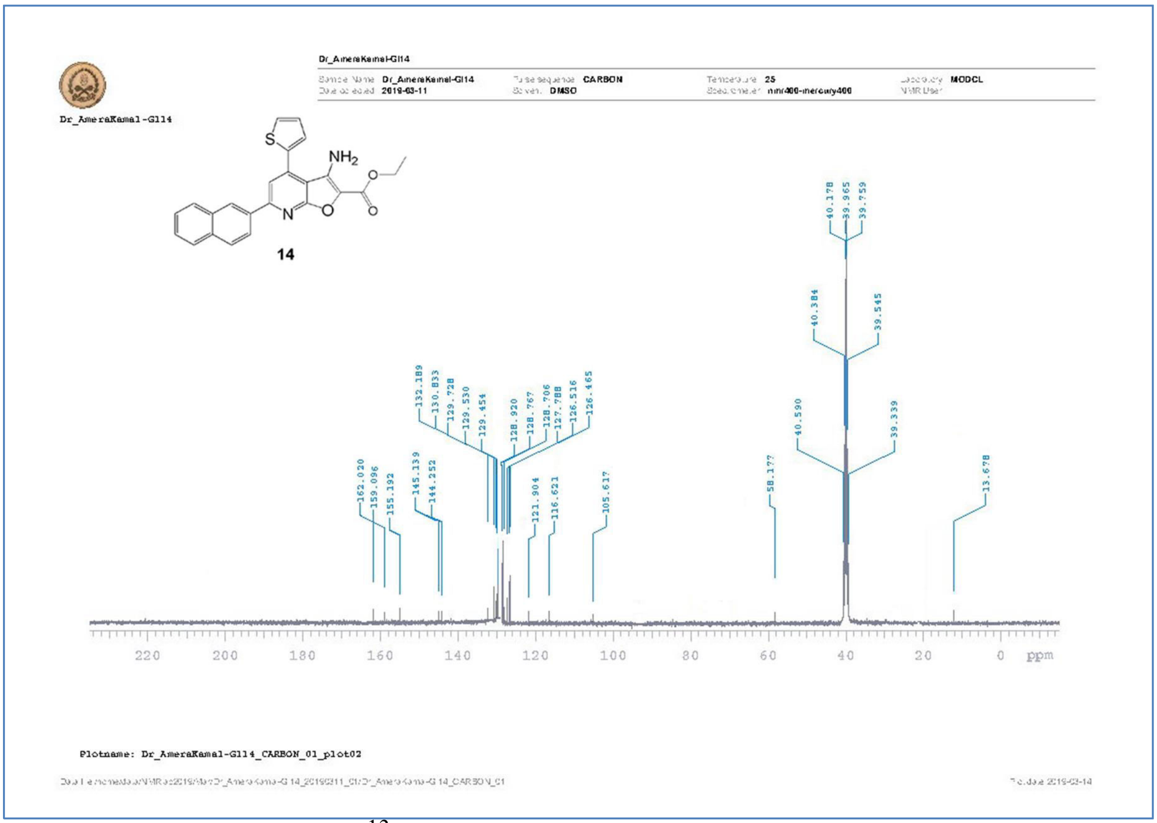

^{13}C NMR of compound **14**

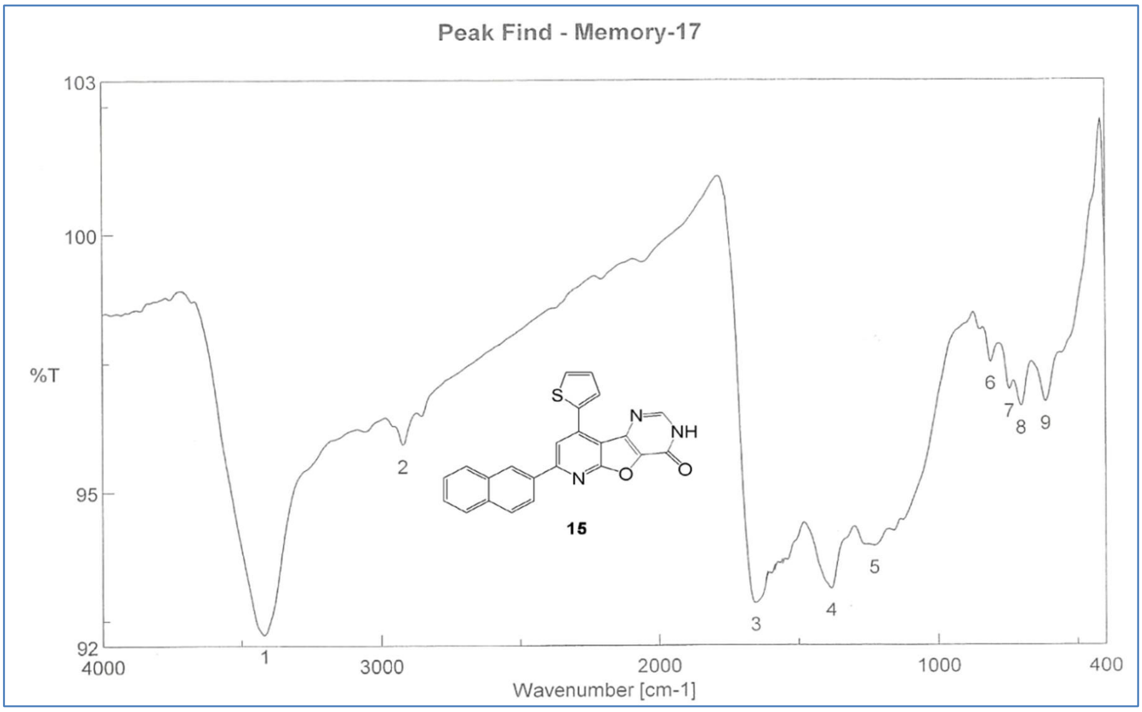

IR spectrum of compound **15**

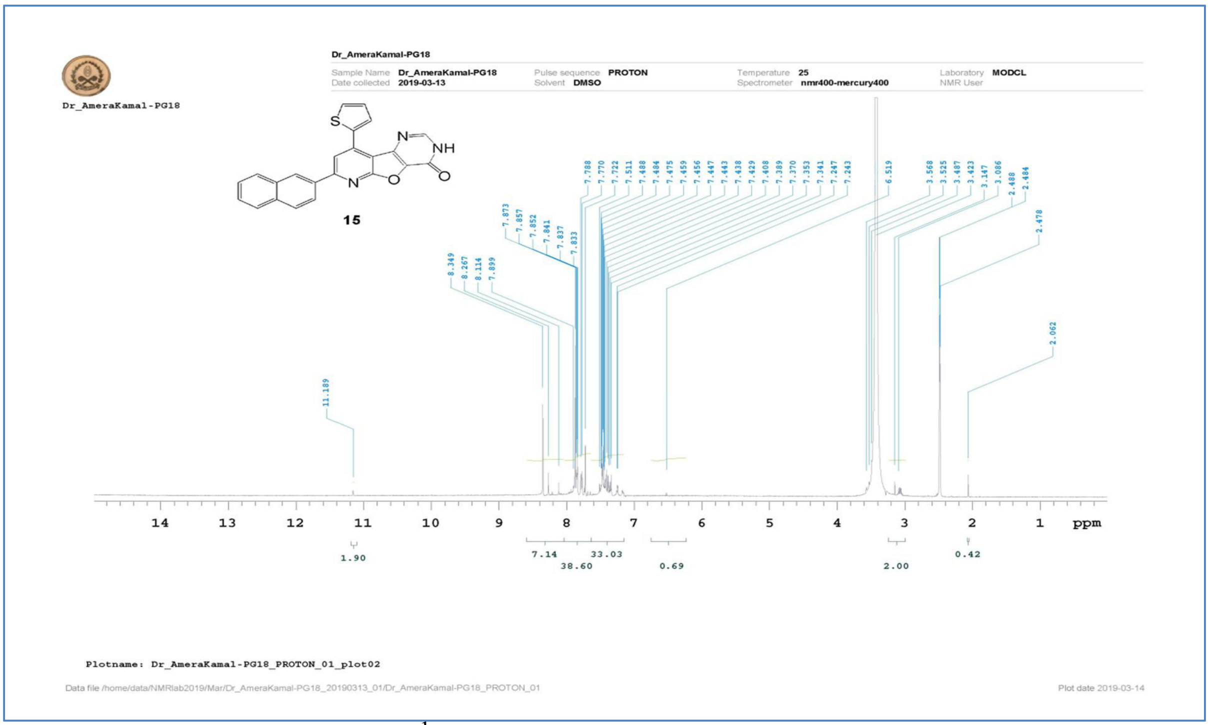

^{1}H NMR of compound **15**
